# Supplementary material for: Intersection of reproductive coercion and intimate partner violence: cross-sectional influences on women’s contraceptive use in Burkina Faso, Côte d’Ivoire and Kenya
Source: BMJ Open. 2023 May 10;13(5):e065697. doi: 10.1136/bmjopen-2022-065697 (PMC10173961; doi:10.1136/bmjopen-2022-065697)
Supplement: Author's manuscript [file bmjopen-2022-065697.draft_revisions.pdf]

BMJ Open

BMJ Open is committed to open peer review. As part of this commitment we make the peer review history of every article we publish publicly available.

When an article is published we post the peer reviewers' comments and the authors' responses online. We also post the versions of the paper that were used during peer review. These are the versions that the peer review comments apply to.

The versions of the paper that follow are the versions that were submitted during the peer review process. They are not the versions of record or the final published versions. They should not be cited or distributed as the published version of this manuscript.

BMJ Open is an open access journal and the full, final, typeset and author-corrected version of record of the manuscript is available on our site with no access controls, subscription charges or pay-per-view fees (http://bmjopen.bmj.com).

If you have any questions on BMJ Open's open peer review process please email info.bmjopen@bmj.com

BMJ Open

The intersection of reproductive coercion and intimate partner violence: influences on women's contraceptive use in Burkina Faso, Côte d'Ivoire, and Kenya

Journal:	*BMJ Open*
Manuscript ID	bmjopen-2022-065697
Article Type:	Original research
Date Submitted by the Author:	16-Jun-2022

Complete List of Authors:	Wood, Shannon; Johns Hopkins University Bloomberg School of Public Health, Department of Population, Family and Reproductive Health; Johns Hopkins University Bloomberg School of Public Health, Bill & Melinda Gates Institute for Population and Reproductive Health<br>Thomas, Haley; Johns Hopkins University Bloomberg School of Public Health, Department of Population, Family and Reproductive Health<br>Thiongo, Mary; International Centre for Reproductive Health Kenya<br>Guiella, Georges; Institut Supérieur des Sciences de la Population<br>Bazié, Fiacre; Institut Supérieur des Sciences de la Population<br>Onadja, Yentéma; Institut Supérieur des Sciences de la Population<br>Mosso, Rosine; Ecole Nationale Superieure de Statistique et d'Economie Appliquee<br>Fassassi, Raimi; Ecole Nationale Superieure de Statistique et d'Economie Appliquee<br>Gichangi, Peter; International Centre for Reproductive Health Kenya; Technical University of Mombasa<br>Decker, Michele; Johns Hopkins University Bloomberg School of Public Health, Population, Family and Reproductive Health

Keywords:	REPRODUCTIVE MEDICINE, PUBLIC HEALTH, GYNAECOLOGY

SCHOLARONE™
Manuscripts

I, the Submitting Author has the right to grant and does grant on behalf of all authors of the Work (as defined in the below author licence), an exclusive licence and/or a non-exclusive licence for contributions from authors who are: i) UK Crown employees; ii) where BMJ has agreed a CC-BY licence shall apply, and/or iii) in accordance with the terms applicable for US Federal Government officers or employees acting as part of their official duties; on a worldwide, perpetual, irrevocable, royalty-free basis to BMJ Publishing Group Ltd ("BMJ") its licensees and where the relevant Journal is co-owned by BMJ to the co-owners of the Journal, to publish the Work in this journal and any other BMJ products and to exploit all rights, as set out in our licence.

The Submitting Author accepts and understands that any supply made under these terms is made by BMJ to the Submitting Author unless you are acting as an employee on behalf of your employer or a postgraduate student of an affiliated institution which is paying any applicable article publishing charge ("APC") for Open Access articles. Where the Submitting Author wishes to make the Work available on an Open Access basis (and intends to pay the relevant APC), the terms of reuse of such Open Access shall be governed by a Creative Commons licence – details of these licences and which Creative Commons licence will apply to this Work are set out in our licence referred to above.

Other than as permitted in any relevant BMJ Author's Self Archiving Policies, I confirm this Work has not been accepted for publication elsewhere, is not being considered for publication elsewhere and does not duplicate material already published. I confirm all authors consent to publication of this Work and authorise the granting of this licence.

For peer review only

The intersection of reproductive coercion and intimate partner violence: influences on women's contraceptive use in Burkina Faso, Côte d'Ivoire, and Kenya

Shannon N. Wood[1,2]
Haley L. Thomas[1]
Mary Thiongo[3]
Georges Guiella[4]
Fiacre Bazié [4]
Yentéma Onadja[4]
Rosine Mosso[5]
Raimi Fassassi[5]
Peter Gichangi[3,6,7]
Michele R. Decker[1,2,8]

[1] Department of Population, Family and Reproductive Health, Johns Hopkins Bloomberg School of Public Health, Baltimore, USA.
[2] Bill & Melinda Gates Institute for Population and Reproductive Health, Department of Population, Family and Reproductive Health, Johns Hopkins Bloomberg School of Public Health, Baltimore, USA.
[3] International Centre for Reproductive Health-Kenya, Nairobi, Kenya.
[4] Institut Supérieur des Sciences de la Population (ISSP/Université Joseph Ki-Zerbo), Ouagadougou, Burkina Faso
[5] Ecole Nationale Superieure de Statistique et d'Economie Appliquee (ENSEA), Abidjan, Cote d'Ivoire.
[6] Technical University of Mombasa, Mombasa, Kenya.
[7] Department of Public Health and Primary Care, Faculty of Medicine and Health Sciences, Ghent University, Belgium
[8] Johns Hopkins School of Nursing, Baltimore, USA.

Corresponding author
Shannon N. Wood
615 N Wolfe St, E4009
Baltimore, MD 21205
swood@jhu.edu

For peer review only

Abstract

Objectives: Among nationally representative cross-sections of women in need of contraception from Burkina Faso, Côte d'Ivoire, and Kenya, we aimed to: 1) examine the intersection of past-year physical/sexual intimate partner violence (IPV), emotional IPV, and reproductive coercion (RC); 2) assess the impact of physical/sexual IPV, emotional IPV, and RC on women's contraceptive use outcomes, including current contraceptive use, method type, and covert use.

Methods: The present analysis uses cross-sectional female data collected in Burkina Faso (December 2020-March 2021), Côte d'Ivoire (October-November 2021) and Kenya (November-December 2020). Analytic samples were restricted to partnered women with contraceptive need who completed a violence module (Burkina Faso n=1,863; Côte d'Ivoire n=1105; Kenya n=3390). The exposures of interest—past-year emotional IPV, physical/sexual IPV, and RC—were assessed using abridged versions of the Revised Conflict and Tactics Scale (CTS-2) and Reproductive Coercion Scale, respectively. Outcomes of interest included current contraceptive use, contraceptive method mix, and covert contraceptive use, and utilized standard assessments. By site, prevalence and overlap of IPV and RC experiences were calculated, and bivariate and multivariable logistic regression used to examine outcomes by violence exposure.

Results: Across sites, $6.4\%_{\text{Côte d'Ivoire}}$ to $7.8\%_{\text{Kenya}}$ of women in need of contraception experienced RC; approximately one-third to one-half of women experiencing RC reported no other violence forms ($31.7\%_{\text{Burkina Faso}}$ to $45.8\%_{\text{Côte d'Ivoire}}$), whereas physical/sexual IPV largely occurred with emotional IPV. In multivariable models, RC was consistently associated with covert use ($aOR_{\text{Burkina Faso}}$=2.84 [95% CI=1.21-6.64]; $aOR_{\text{Côte d'Ivoire}}$=4.45 [95% CI=1.76-11.25]; aOR_{Kenya}=5.77 [95% CI=3.51-9.46]. Some IPV in some settings was also associated with covert use (emotional IPV $aOR_{\text{Burkina Faso}}$=2.99; 95% CI=1.56-5.74; physical/sexual aOR_{Kenya}=2.35; 95% CI=1.33-4.17).

Conclusions: Across settings, covert use is a critical strategy for women experiencing RC. Country policies must recognize RC as a unique form of violence with profound implications for women's reproductive health.

For peer review only - http://bmjopen.bmj.com/site/about/guidelines.xhtml

Key Messages

- **What is already known on this topic** – Ambiguity remains surrounding how reproductive coercion (RC) and intimate partner violence (IPV) may uniquely impact reproductive health outcomes, including contraceptive use.
- **What this study adds** – While many physical/sexual IPV experiences interplayed with other forms of violence, the majority of women experiencing RC experienced this form of abuse in isolation or coupled with emotional IPV. Across distinct contexts in East and West Africa, RC was associated with three- to five-fold heightened odds of covert use of contraception; associations between physical/sexual IPV and emotional IPV and contraceptive use outcomes were less consistent.
- **How this study might affect research, practice or policy** – To counteract RC's adverse reproductive health impact, synergy is needed between family planning and violence services, including universal and judgement-free provision of affordable covert contraceptive methods.

For peer review only

Introduction

Intimate partner violence (IPV) is pervasive globally—recent evidence highlights that 27% of women are impacted by physical or sexual IPV.[1] Partners can further hinder women's health and livelihoods through psychological and economic abuse and coercive control.[2,3] Reproductive coercion (RC), or partner control over contraceptive use and reproductive health trajectories,[4,5] was initially conceptualized as a unique form of violence that could occur in tandem with physical or sexual IPV.[6,7] RC,[6,7] like IPV,[8] has a negative impact on women's reproductive health trajectories and has been linked to decreased use of effective contraception and increased risk of unintended pregnancy.

Current evidence examining the reproductive health impact of these leading forms of abuse has been concentrated within high-income contexts, namely, the United States[6,7] and Australia.[9] Recent literature, however, highlights that RC is also common for sub-Saharan African women, though behaviors, intentions, and motivations may differ in comparison to high-income contexts.[10–13] Specifically, partners may enact a broader range of RC behaviors with the means of asserting power over reproductive decision-making, including seeking to prevent pregnancy against a woman's wishes.[10]

Understanding the impact of abusive partner dynamics on women's reproductive health in sub-Saharan Africa is critical given that sub-Saharan African women bear a disproportionate adverse health burden, specifically the highest global rates of unintended pregnancy,[14] maternal mortality,[15] and unsafe abortion.[16] Multi-country studies have sought to understand the impact of IPV on contraceptive use with inconclusive findings.[17] Studies have reported increased contraceptive and covert use among IPV survivors,[12] whereas others have indicated decreased contraceptive use.[18] Moreover, such studies have largely failed to disentangle the impact of RC and emotional abuse on women's reproductive health outcomes, though women may be pressured or shamed when seeking to delay or limit childbearing; as such, these forms of violence may significantly impact contraceptive use and continuation.[11,13,17]

Inclusion of comprehensive RC measures within recent population-based surveys allows for the examination of the interplay of violence experiences and their impact on contraceptive use outcomes. To date, analyses examining linkages between unique forms of violence and contraceptive use have been limited given lack of RC and IPV measures simultaneously included in national surveys. In three national samples across East and West Africa, this study aimed to: 1) examine the intersection of physical/sexual IPV, emotional IPV, and RC and 2) assess the impact of IPV and RC on women's contraceptive use outcomes (current contraceptive use, method type, and covert use).

Methods

Study Contexts

In 2019, Burkina Faso, Côte d'Ivoire, and Kenya ranked similarly on the United Nations Development Program (UNDP) Gender Inequality Index (Burkina Faso 0.594, rank 147 out of 162;[19] Côte d'Ivoire 0.638, rank 153;[20] Kenya 0.518, rank 126).[21] All three countries have ratified the Convention on the Elimination of all Forms of Discrimination Against Women (CEDAW) and

For peer review only - http://bmjopen.bmj.com/site/about/guidelines.xhtml

have taken steps to protect women from gender-based violence (GBV),[22] however, RC has not been formally included as form of violence within national policies.

Overview of PMA and GBV Module

Analyses utilize data from Performance Monitoring for Action (PMA), which administers annual population-based panel and cross-sectional questionnaires at the household, female, and facility levels using a multi-stage cluster design allowing for nationally and regionally representative estimates. Additional details are available at pmadata.org.

This analysis uses cross-sectional female data collected in Burkina Faso (December 2020-March 2021), Côte d'Ivoire (October-November 2021) and Kenya (November-December 2020). Sites were selected due to implementation of the PMA GBV module.

All female household members ages 15-49 were eligible for the PMA female survey. In accordance with ethical best practices,[23,24] only one female per household was eligible for the GBV module. If there was more than one eligible female per household, one woman per household was randomly selected.

Training and Ethical Protections

Data were collected by trained resident enumerators (REs) using mobile phones equipped with Open Data Kit software; privacy checks were built into the survey. Prior to implementation, all REs completed a GBV-specific training, which emphasized maintaining confidentiality and privacy, asking non-judgmental questions, monitoring for emotional upset, and referring women to support services. In line with best practices,[23,24] all participants, regardless of violence disclosure or selection into the module, received a referral sheet for GBV and health resources.

All participants provided oral consent to participate. Approval was received by ethical review committees at Johns Hopkins School of Public Health, Comite D'Ethique Pour La Recherche en Sante, Ministere de la Recherche Scientifique et de L'Innovation, Ministere de la Sante in Burkina Faso, Comité National d'Ethique des Sciences de la Vie et de la Santé (CNESVS) in Côte d'Ivoire, and Kenyatta National Hospital-University of Nairobi Ethics and Research Committee College of Health Sciences in Kenya.

Patient and Public Involvement

The GBV module was included within select PMA surveys at the request of the Ministries of Health. Results are disseminated to in-country stakeholders at national and local events.

Analytic Samples

One woman per household was randomly selected to complete the GBV module (n=4,289 Burkina Faso; n=2,752 Côte d'Ivoire; n=6,870 Kenya); participants did not complete the GBV module if there were privacy concerns (n=60 Burkina Faso; n=88 Côte d'Ivoire; n=119 Kenya). IPV and RC items were only administered to those married or partnered, for a final sample of 3,048 women in Burkina Faso, 1,852 women in Côte d'Ivoire, and 4,355 women in Kenya.

The analytic sample was further restricted to women in need of contraception, defined as being sexually active in the last 12 months, wanting to wait more than 12 months to have another child

For peer review only - http://bmjopen.bmj.com/site/about/guidelines.xhtml

or not wanting any more children, not currently pregnant, and fecund, for a final sample of n=1,863 Burkina Faso, n=1,105 Côte d'Ivoire, n=3,390 Kenya. Analytic samples float to accommodate small amounts of missing data (<1%).

Measures

Three independent variables classified past-year experiences of violence (emotional IPV, physical/sexual IPV, and RC) via behavioral assessment. Past-year IPV was measured via 5-item abbreviated version of the Revised Conflict and Tactics Scale (CTS-2).[25] Specifically, emotional IPV was assessed via affirmative response to a single behavior (insulted you, yelled at you, screamed or made humiliating remarks). Physical/sexual IPV was assessed via affirmative response to any of the following behaviors: slapped, hit, or physically hurt you; threatened with a weapon or attempted to strangle or kill you; pressured or insisted on having sex when you did not want to (without physical force); physically forced you to have sex when you did not want to. Past-year RC was measured via 6-items from the pregnancy coercion sub-scale of the Reproductive Coercion Scale[26] and previously adapted to the sub-Saharan African context.[12]

Three dichotomous dependent contraceptive use variables (current contraceptive use, method classification [female-controlled or male compliant] and covert contraceptive use) were examined using standard assessments.[27] For categorization of female-controlled method use, current contraceptive users were asked to identify all contraceptive methods being used; the respondent's most effective method was then categorized as either a male compliant method (male sterilization, male condom, female condom, diaphragm, foam/jelly, standard days/cycle beads, rhythm, withdrawal, other traditional) or a female-controlled method (female sterilization, implant, IUD, injectables, pills, EC, LAM). Direct covert use assessment was utilized.[28]

Several sociodemographic and relationship characteristics were explored as potential confounders between violence and contraceptive use outcomes using standard assessments,[27] including residence (urban/rural); parity (0, 1-2, 3+), marital status (married/living with a partner), polygyny (yes/no), and employment (yes/no).

Statistical Analysis

All analyses were stratified by site. Sociodemographic, relationship, and sexual and reproductive health characteristics were examined overall and among those with contraceptive need. The prevalence of past-year RC was calculated overall and by item; prevalence of past-year IPV was calculated overall and by IPV sub-type (emotional, physical/sexual). Overlapping experiences of emotional IPV, physical/sexual IPV, and RC were examined via Venn diagrams. Bivariate and multivariable logistic regression models were used to separately examine each contraceptive outcome by experience of violence; specifically, three models were utilized: 1) bivariate; 2) adjustment for sociodemographic and characteristics only; 3) adjustment for sociodemographic and relationship characteristics and other types of violence. Sociodemographic and relationship characteristics were examined for multi-collinearity, and ultimately, the third model was retained given <10% difference in effect estimates between models. All analyses were conducted in Stata version 16 (College Station, TX) and weighted to account for survey design.

Results

For peer review only - http://bmjopen.bmj.com/site/about/guidelines.xhtml

In Burkina Faso and Kenya, most women lived within rural areas, whereas in Côte d'Ivoire, residence was split between urban and rural localities (Table 1). Approximately 90% of partnered Burkinabe and Kenyan women were married compared to large proportions of women from Côte d'Ivoire lived with a partner outside of marriage (39.4%). Polygynous unions were highest in Burkina Faso (28.2%) and lowest in Kenya (11.4%). More than three-quarters of women in need of contraception were currently using contraception in Kenya (77.6%), whereas this proportion was substantially lower in Burkina Faso (52.6%) and Côte d'Ivoire (49.2%). In Kenya and Côte d'Ivoire, short-acting hormonal methods were the most used contraceptive methods (48.4% and 49.9%, respectively); in Burkina Faso, higher proportions of women used LARCs (45.5%). Approximately 11% of contraceptive users in Burkina Faso and Côte d'Ivoire used covertly; this proportion was slightly lower in Kenya (7.6%).

Table 1. Demographic Characteristics of Partnered Women Selected for GBV Module, by Country

	Burkina Faso		Côte d'Ivoire		Kenya	
	Partnered women (n=3048)	FP need (n=1863)	Partnered women (n=1852)	FP need (n=1105)	Partnered women (n=4355)	FP need (n=3390)
	%*					
**Sociodemographic**						
Residence						
Urban	19.6	20.2	48.5	47.7	30.6	30.8
Rural	80.4	79.9	51.5	52.3	69.4	69.2
Household Wealth Tertile						
Lowest	35.1	37.4	39.1	39.6	37.0	36.7
Middle	33.8	32.5	31.0	31.3	33.6	33.8
Highest	31.1	30.1	29.9	29.1	29.5	29.5
Household composition						
Does not live with extended family	63.7	64.0	57.7	59.1	76.1	76.3
Lives with extended family	36.3	36.0	42.3	41.0	23.9	23.7
Age						
15-19	7.5	7.6	5.3	5.5	2.1	1.8
20-29	42.0	43.3	36.1	38.8	38.7	37.9
30-39	34.0	32.9	40.9	41.6	39.0	40.6
40-49	16.4	16.2	17.8	14.1	20.3	19.7
Education						
None	67.0	67.6	52.6	49.5	4.8	4.1
Primary	18.5	18.2	26.6	27.1	51.4	52.0
Secondary or Higher	14.5	14.2	20.7	23.4	43.9	43.9
Parity						
0	4.7	0.8	6.4	1.6	3.7	1.4
1-2	33.2	33.8	35.3	33.3	37.9	36.8
3+	62.2	65.4	58.3	65.2	58.5	61.8
**Relationship Dyad**						

For peer review only - http://bmjopen.bmj.com/site/about/guidelines.xhtml

Marital status						
Married	90.9	92.4	64.2	60.6	89.5	89.6
Living with partner	9.1	7.6	35.9	39.4	10.5	10.4
Polygamous union	30.5	28.2	18.4	16.6	12.1	11.4
Age at marriage						
≦15	5.3	4.9	11.9	11.8	7.0	7.1
>15 & <18	47.0	46.9	29.1	31.0	22.6	22.0
≧18	47.8	48.2	59.0	57.2	70.4	70.9
Partner education						
None	62.5	63.4	40.3	39.1	3.9	3.4
Primary	21.2	21.0	24.0	22.7	44.7	45.6
Secondary or Higher	16.3	15.6	35.7	38.2	51.4	51.0
**Finance**						
Employed	29.8	30.3	47.1	47.8	48.0	49.3
Has savings	15.3	15.4	8.7	8.7	48.0	48.1
Level of financial knowledge						
Not at all or not very	92.9	92.8	63.7	63.7	21.8	21.7
Somewhat or very	7.1	7.2	36.3	36.3	78.2	78.3
Economically reliant on partner for basic needs	51.8	52.4	56.2	56.4	61.9	61.4
**SRH**						
Currently pregnant	10.4	--	10.3	--	8.1	--
Feeling when found out about pregnancy[1]						
Very/sort of happy	74.5	--	67.2	--	66.9	--
Mixed happy and unhappy	1.8	--	7.3	--	12.9	--
Sort of/very unhappy	23.7	--	25.6	--	20.2	--
Currently using any contraceptives	36.0	52.6	32.8	49.2	66.1	77.6
Method mix among users[2]						
Male Compliant	8.0	8.2	7.8	7.7	3.5	3.5
LARC†	45.7	45.5	19.8	19.5	41.5	39.4
Short-acting hormonal	35.8	35.9	48.6	49.9	46.5	48.4
Traditional	10.6	10.4	23.7	22.9	8.5	8.8
Covert contraceptive use	12.5	11.1	12.0	11.3	7.9	7.6
Feeling if you got pregnant now[3]						
Very/sort of happy	44.3	33.7	50.4	34.8	37.3	32.5
Mixed happy and unhappy	4.9	5.9	8.2	10.8	16.0	17.9
Sort of/very unhappy	50.8	60.4	41.4	54.4	46.6	49.7

[1] Among women who are currently pregnant
[2] Male compliant (male sterilization, male and female condoms, diaphragm, foam); LARC (implant, IUD, female sterilization); short-acting hormonal (pill, injectables, n-tablet, EC); traditional (cycle beads, LAM, rhythm method, withdrawal, other traditional)
[3] Among women who are not currently pregnant
† Includes female sterilization (n=4 in Burkina Faso, n=0 in Côte d'Ivoire, n=102 in Kenya)

Across sites, emotional IPV was the most prevalent type of violence experienced among women in need of contraception (20.1% Kenya, 23.9% Burkina Faso, 29.0% Côte d'Ivoire), followed by physical/sexual IPV (9.9% Burkina Faso, 13.2% Kenya, 14.5% Côte d'Ivoire) and RC (6.4% Côte d'Ivoire, 7.0% Burkina Faso, 7.8% Kenya; Table 2). Mistreatment for wanting to use family planning was the most common RC behavior (5.2% Burkina Faso, 4.4% Côte d'Ivoire, and 5.3% Kenya), whereas physical violence as a result of not getting pregnant was rarer (0.3% Burkina Faso, 0.4% Côte d'Ivoire, 1.2% Kenya).

Table 2. Prevalence of Past-Year Reproductive Coercion and Intimate Partner Violence among Partnered Women with Need for Contraception, by Country

	**Burkina Faso**	**Côte d'Ivoire**	**Kenya**
	% weighted		
Individual RC Items			
Mistreated you for wanting to use family planning	5.2	4.4	5.3
Forced or pressured you to become pregnant	2.2	3.2	4.3
Said he would leave if you did not get pregnant	1.3	1.6	1.9
Said he would have baby with someone else if you did not get pregnant	1.3	1.9	1.9
Took away your family planning or prevented you from going to clinic for family planning	1.6	0.9	2.0
Hurt you physically because you did not get pregnant	0.3	0.4	1.2
Any Experience of RC	7.0	6.4	7.8
IPV			
**Emotional IPV**	23.9	29.0	20.1
**Physical/sexual IPV**	9.9	14.5	13.2
Any Experience of IPV	26.9	32.1	23.1

Women in need of contraception experienced multiple types of violence simultaneously, with 10.1%, 12.9%, and 12.7% experiencing two of more types of violence in Burkina Faso, Côte d'Ivoire, and Kenya respectively (Figure 1). When exclusively examining reports of RC (n=139 Burkina Faso; n=72 Côte d'Ivoire; n=251 Kenya), approximately one-third to one-half of women reported RC experience in isolation (31.7% (44/139) Burkina Faso, 45.8% (33/72) Côte d'Ivoire, 35.1% (88/251) Kenya). In Burkina Faso, substantial portions women experiencing RC reported RC in tandem with emotional IPV (36.0% (50/139)), whereas in Côte d'Ivoire and Kenya, approximately one-third reported overlap with both emotional IPV and physical/sexual IPV (36.1% (26/72) and 31.9% (80/251), respectively).

RC was consistently associated with increased covert use of contraception across sites (Burkina Faso: aOR=2.84; 95% CI=1.21-6.64; Côte d'Ivoire: aOR=4.45; 95% CI=1.76-11.25; Kenya: aOR=5.77; 95% CI=3.51-9.46; Table 3). Emotional IPV was associated further with covert

contraceptive use in Burkina Faso (aOR=2.99; 95% CI=1.56-5.74) and physical/sexual IPV with covert contraceptive use in Kenya (aOR=2.35; 95% CI=1.33-4.17). In Kenya, emotional IPV was associated with increased odds of current contraceptive use (aOR=1.44; CI=1.04-2.01), and in Burkina Faso, emotional IPV was marginally associated with current contraceptive use below the significance threshold (aOR=1.36; 95% CI=0.97-1.92; p=0.08). Also, in Burkina Faso, RC experience was associated with non-significant decreases in current contraceptive use (aOR=0.66; 95% CI=0.43-1.01; p=0.06). None of the violence forms displayed significant associations with method type.

For peer review only

Table 3. Logistic Regression between Past-Year RC and IPV and Contraceptive Use Outcomes Among Partnered Women in Need of Contraception, per Country

**Burkina Faso**									
	Reproductive Coercion			Emotional IPV			Physical/ Sexual IPV		
	No RC (n=1719)	RC (n=139)	aOR[œ]	No IPV (n=1332)	IPV (n=529)	aOR[œ]	No IPV (n=1686)	IPV (n=174)	aOR[œ]
	%			%			%		
Current contraceptive use	53.2	46.2	0.66 (0.43, 1.01) ±	50.9	**58.2***	1.36 (0.97, 1.92) ±	52.6	52.7	0.91 (0.54, 1.54)
Method mix, among users									
Male compliant[1]	18.5	19.5	ref	19.5	15.9	ref	19.0	15.0	ref
Female-controlled[2]	81.5	80.6	0.81 (0.36, 1.86)	80.5	84.1	1.23 (0.72, 2.09)	81.1	85.0	1.14 (0.47, 2.73)
Covert contraceptive use	9.9	**29.3*****	**2.84 (1.21, 6.64)***	7.5	**20.9*****	**2.99 (1.56, 5.74)*****	10.3	17.9	0.83 (0.36, 1.91)
**Côte d'Ivoire**									
	RC			Emotional IPV			Physical/ Sexual IPV		
	No RC (n=1026)	RC (n=72)	aOR[œ]	No IPV (n=778)	IPV (n=327)	aOR[œ]	No IPV (n=940)	IPV (n=165)	aOR[œ]
	%			%			%		
Current contraceptive use	49.1	54.2	1.30 (0.72, 2.34)	48.1	52.0	1.15 (0.67, 1.97)	48.2	54.9%	1.25 (0.61, 2.56)
Method mix, among users									
Male compliant	30.7	19.3	ref	31.4	26.3	ref	30.8	24.5	ref
Female-controlled	69.3	80.7	--	68.7	73.7	1.20 (0.67, 2.14)	69.2	75.5	--

For peer review only

	RC			Emotional IPV			Physical/ Sexual IPV		
Covert contraceptive use	9.5	**35.0*****	**4.45 (1.76, 11.25)****	9.4	15.5	1.88 (0.87, 4.05)	10.9	13.0	0.59 (0.19, 1.86)
**Kenya**									
	RC			Emotional IPV			Physical/ Sexual IPV		
	No RC (n=3135)	RC (n=251)	aOR[œ]	No IPV (n=2741)	IPV (n=646)	aOR[œ]	No IPV (n=2986)	IPV (n=401)	aOR[œ]
	%			%			%		
Current contraceptive use	77.9	74.2	0.84 (0.57, 1.23)	77.2	79.2	**1.44 (1.04, 2.01)***	77.8	76.3	0.82 (0.60, 1.13)
Method mix, among users									
Male compliant	10.7	12.1	ref	10.3	12.6	ref	10.6	11.5	ref
Female-controlled	89.4	87.9	0.97 (0.55, 1.69)	89.7	87.4	0.73 (0.50, 1.05)±	89.4	88.5	1.18 (0.67, 2.07)
Covert contraceptive use	5.6	**30.8*****	**5.77 (3.51, 9.46)*****	6.1	**13.2*****	0.8 (0.45, 1.42)	6.1	**17.5*****	**2.35 (1.33, 4.17)****

±<0.1; *<0.05; **<0.01; ***<0.001; bolding represents significance p<0.05
œaOR adjusted for other forms of violence, residence, education, parity, marital status, polygyny, employment
-- cells n<10 suppressed for regression
Male compliant: Male sterilization, male condom, female condom, diaphragm, foam/jelly, standard days/cycle beads, rhythm, withdrawal, other traditional; Female-controlled: Female sterilization, implant, IUD, injectables, pills, EC, LAM

For peer review only - http://bmjopen.bmj.com/site/about/guidelines.xhtml

Discussion

Leveraging large-scale, population-based samples, this analysis illuminates the prevalence of RC and overlap with physical/sexual and emotional IPV, and its impact on current and covert contraceptive use in Burkina Faso, Côte d'Ivoire, and Kenya. Data illustrate that for women experiencing RC who are in need of contraception, RC often occurred in isolation (31.7%$_{\text{Burkina Faso}}$ to 45.8%$_{\text{Côte d'Ivoire}}$) or coupled with emotional IPV (16.7%$_{\text{Côte d'Ivoire}}$ to 36.0%$_{\text{Burkina Faso}}$). Accordingly, only situating RC interventions within IPV programs and response services may overlook the needs of women who are experiencing this unique, yet detrimental, form of abuse. Further, screening for physical or sexual IPV alone will fail to capture most RC experiences.

Notably, experience of past-year RC was consistently associated with increased covert use of contraception across sites (Burkina Faso: aOR=2.84; 95% CI=1.21-6.64; Côte d'Ivoire: aOR=4.45; 95% CI=1.76-11.25; Kenya: aOR=5.77; 95% CI=3.51-9.46), however, similar results were not observed for female-controlled method use. Covert use may prove a promising, yet risky safety strategy for women seeking to avert pregnancy and also experiencing RC.[12] Qualitative data with IPV and RC survivors in Nairobi indicated the cyclic covert use experiences of women—first electing to use contraception covertly given RC, then IPV or RC upon partner finding out about use, followed by a subsequent attempt to use a different contraceptive method covertly.[29] While providers may recommend female-controlled methods in light of partner disapproval, such as IUDs or implants, control of method use alone may not be enough to protect against unintended pregnancy when faced with RC. Instead, providers must be aware of RC and inquire about covert use preferences and potential RC experiences during contraceptive counseling to ensure method continuity and aversion of pregnancy without detriment to women's safety.

Consistent with Demographic and Health Survey data, associations between physical/sexual IPV and contraceptive use were statistically insignificant,[17] however, emotional IPV was associated with significant increases in current contraceptive use in Kenya (aOR=1.44; 95% CI=1.04; p<0.05) and trend towards association in Burkina Faso (aOR=1.36; 95% CI=0.97-1.92; p=0.06). These results may signify important benchmarks on the abuse cycle and pathways to help-seeking—women wishing to avert pregnancy within violent relationships are acting on their reproductive preferences to utilize contraception. Further, they justify the inclusion of emotional abuse measures within large-scale surveys given its high prevalence and concurrence with RC.

Results must be considered in light of limitations. Foremost, findings are cross-sectional, limiting conclusions surrounding temporality of associations—longitudinal work is needed to disentangle whether women are using contraception because of their abuse or whether the abuse is incurred given contraceptive use. Further, RC measures did not account for abortion coercion experiences or condom use manipulation, thus may undercount the true burden of RC in these contexts. Social desirability biases and privacy concerns could further contribute to underreporting of abuse, despite extensive training and privacy protocols aligned with best practices.[23,24]

Importantly, policymakers and family planning and violence service providers must recognize RC and its health impact to holistically support women's needs. Work to dismantle deep-rooted social norms that dictate spousal permission prior to contraceptive provision must be included in

healthcare provider trainings on how to support women experiencing RC. Interventions such as ARCHES in Kenya[30] and have proven useful in decreasing RC through training family planning service providers to recognize RC as a prevalent form of abuse that impacts women's reproductive health[30]—scale-up of such programs may help prevent recurrent abuse. Integration of violence and reproductive health prevention and response services can further guarantee that women's needs are met regardless of type of abuse incurred. Standardization of recommendations can further ensure that women are able to enact safety strategies to protect their reproductive health while concurrently mitigating the impact of abuse. Though many countries have made recent notable steps towards GBV prevention and response, national policies do not currently recognize RC. Policies must not only name RC as a detriment to women's health, but also include practical rights-based solutions that ensure women's privacy in contraceptive decision-making and freedom from partner interference, such as universal, affordable, and judgement-free provision of covert contraceptive methods and emergency contraception, to ultimately counteract its reproductive health impact.

For peer review only

Ethics approval statement

Institutional Review Board approval was obtained at JHSPH (00009677), Comité d'Ethique pour la recherche en santé and the Ministère de la Santé et Ministère de l'Enseignement Supérieur, de la Recherche Scientifique et de l'Innovation (ISSP/DA/GG/062/2021), Comité d'Ethique de la Recherche Institut Pasteur de la Côte d'Ivoire (150-21/MSHP/CNESVS-km), and Kenya Medical Research Institute (KEMRI) Ethics Review Committee (KNH-ERC/Mod&SAE/314).

Contributorship statement

SNW: study design, oversight, analysis, initial draft. HLT: analysis, graphics, initial draft. MT, GG, FB, YO, RM, RF, PG: study design, oversight of data collection, ethical adherence, interpretation, and editing. MRD: study design, oversight, initial draft. All authors participated in writing and approving the final manuscript.

Competing interests

None declared.

Funding

This work was supported, in whole, by the Bill & Melinda Gates Foundation [009639]. Under the grant conditions of the Foundation, a Creative Commons Attribution 4.0 Generic License has already been assigned to the Author Accepted Manuscript version that might arise from this submission.

Data availability statement

Data are available upon request from pmadata.org.

For peer review only

References

1. Sardinha L, Maheu-Giroux M, Stöckl H, Meyer SR, García-Moreno C. Global, regional, and national prevalence estimates of physical or sexual, or both, intimate partner violence against women in 2018. *Lancet*. 2022;399(10327):803-813. doi:10.1016/S0140-6736(21)02664-7
2. Stylianou AM. Economic abuse within intimate partner violence: A review of the literature. *Violence Vict*. 2018;33(1):3-22. doi:10.1891/0886-6708.VV-D-16-00112
3. Heise L, Pallitto C, García-Moreno C, Clark CJ. Measuring psychological abuse by intimate partners: Constructing a cross-cultural indicator for the Sustainable Development Goals. *SSM - Popul Heal*. 2019;9(September 2018):1-6. doi:10.1016/j.ssmph.2019.100377
4. Silverman JG, Raj A. Intimate Partner Violence and Reproductive Coercion : Global Barriers to Women's Reproductive Control. *Plos Med*. 2014;11(9):9-12. doi:10.1371/journal.pmed.1001723
5. Grace KT, Anderson JC. Reproductive Coercion. *Trauma, Violence, Abus*. Published online 2016:1-20. doi:10.1177/1524838016663935
6. Miller E, Decker MR, McCauley HL, et al. Pregnancy coercion, intimate partner violence and unintended pregnancy. *Contraception*. 2010;81(4):316-322. doi:10.1016/j.contraception.2009.12.004
7. Miller E, Decker MR, McCauley HL, et al. A family planning clinic partner violence intervention to reduce risk associated with reproductive coercion. *Contraception*. 2011;83(3):274-280. doi:10.1016/j.contraception.2010.07.013
8. Bergmann JN, Stockman JK. How does intimate partner violence affect condom and oral contraceptive use in the United States ? A systematic review of the literature. *Contraception*. 2015;91:438-455.
9. Tarzia L, Srinivasan S, Marino J, Hegarty K. Exploring the gray areas between "stealthing" and reproductive coercion and abuse. *Women Heal*. 2020;60(10):1174-1184. doi:10.1080/03630242.2020.1804517
10. Wood SN, Kennedy SR, Akumu I, et al. Reproductive Coercion among Intimate Partner Violence Survivors in Nairobi. *Stud Fam Plann*. 2020;51(4):343-360.
11. Wood SN, Dozier J, Karp C, et al. Pregnancy Coercion, Correlates, and Associated Modern Contraceptive Use Within a Nationally Representative Sample of Ethiopian Women. In: *Population Association of America Annual Meeting*. ; 2021.
12. Silverman JG, Challa S, Boyce SC, Averbach S, Raj A. Associations of reproductive coercion and intimate partner violence with overt and covert family planning use among married adolescent girls in Niger. *EClinicalMedicine*. 2020;22(100359).
13. Boyce SC, Uysal J, Delong SM, et al. Women's and girls' experiences of reproductive coercion and opportunities for intervention in family planning clinics in Nairobi, Kenya: A qualitative study. *Reprod Health*. 2020;17(1):1-12. doi:10.1186/s12978-020-00942-7
14. Darroch JE, Singh S. Trends in contraceptive need and use in developing countries in 2003, 2008, and 2012: An analysis of national surveys. *Lancet*. 2013;381(9879):1756-1762. doi:10.1016/S0140-6736(13)60597-8
15. Alkema L, Chou D, Hogan D, et al. Global, regional, and national levels and trends in maternal mortality between 1990 and 2015, with scenario-based projections to 2030: a systematic analysis by the UN Maternal Mortality Estimation Inter-Agency Group. *Lancet*. 2016;387(10017):462-474. doi:10.1016/S0140-6736(15)00838-7
16. Tsui AO, McDonald-Mosley R, Burke AE. Family planning and the burden of unintended

For peer review only - http://bmjopen.bmj.com/site/about/guidelines.xhtml

pregnancies. *Epidemiol Rev*. 2010;32(1):152-174. doi:10.1093/epirev/mxq012
17. Macquarrie KLD, Mallick L, Kishor S. *DHS Analytical Studies 57: Intimate Partner Violence and Interruption to Contraceptive Use*.; 2016.
18. Maxwell L, H B, Ndyanabo A, et al. The impact of intimate partner violence on women's contraceptive use: Evidence from the Rakai Community Cohort Study in Rakai, Uganda. *Soc Sci Med*. 2018;209(April):25-32. doi:10.1016/j.socscimed.2018.04.050 LK - http://vb3lk7eb4t.search.serialssolutions.com?sid=EMBASE&issn=18735347&id=doi:10.1016%2Fj.socscimed.2018.04.050&atitle=The+impact+of+intimate+partner+violence+on+women%27s+contraceptive+use%3A+Evidence+from+the+Rakai+Community+Cohort+Study+in+Rakai%2C+Uganda&stitle=Soc.+Sci.+Med.&title=Social+Science+and+Medicine&volume=209&issue=&spage=25&epage=32&aulast=Maxwell&aufirst=Lauren&auinit=L.&aufull=Maxwell+L.&coden=SSMDE&isbn=&pages=25-32&date=2018&auinit1=L&aui
19. United Nations Development Programme. *Human Development Report 2020: The Next Frontier Human Development and the Anthropocene: Burkina Faso*.; 2020.
20. United Nations Development Programme. *Human Development Report 2020: The Next Frontier Human Development and the Anthropocene: Cote d'Ivoire*.; 2020.
21. United Nations Development Programme. *Human Development Report 2020: The Next Frontier Human Development and the Anthropocene: Kenya*.; 2020.
22. United Nations. Ratification Status for CEDAW-Convention on the Elimination of All Forms of Discrimination against Women. UN Treaty Body Database. Published 2022. Accessed April 4, 2022. https://tbinternet.ohchr.org/_layouts/15/TreatyBodyExternal/Treaty.aspx?Treaty=CEDAW&Lang=en
23. WHO. *Putting Women First: Ethical and Safety Recommendations for Research on Domestic Violence Against Women*.; 1999.
24. World Health Organization (WHO). *Ethical and Safety Recommendations for Intervention Research on Violence against Women*.; 2016.
25. Straus M, Hamby S, Boney-McCoy S, Sugarman D. The Revised Conflict Tactics Scales (CTS2). *J Fam Issues*. 1996;17(3):283-316.
26. McCauley HL, Silverman JG, Jones KA, et al. Psychometric properties and refinement of the Reproductive Coercion Scale. *Contraception*. 2017;95(3):292-298. doi:10.1016/j.contraception.2016.09.010
27. DHS. Demographic and Health Surveys: Model Woman's Questionnaire. 2016;(1):1-75.
28. Choiriyyah I, Becker S. Measuring Women's Covert Use of Modern Contraception in Cross-Sectional Surveys. *Stud Fam Plann*. 2018;49(2):143-157. doi:10.1111/sifp.12053
29. Wood SN, Kennedy SR, Akumu I, et al. Understanding the link between reproductive coercion and covert use of contraception as a safety strategy for women experiencing violence in Nairobi's urban informal settlements. *Violence Against Women Accept Press*. Published online 2022.
30. Uysal J, Carter N, Johns N, et al. Protocol for a matched-pair cluster control trial of ARCHES (Addressing Reproductive Coercion in Health Settings) among women and girls seeking contraceptive services from community-based clinics in Nairobi, Kenya. *Reprod Health*. 2020;17(1):1-14. doi:10.1186/s12978-020-00916-9

For peer review only - http://bmjopen.bmj.com/site/about/guidelines.xhtml

Figure 1. Venn Diagrams of IPV and Reproductive Coercion Experiences, Among Partnered Women with Need for Contraception, by Country

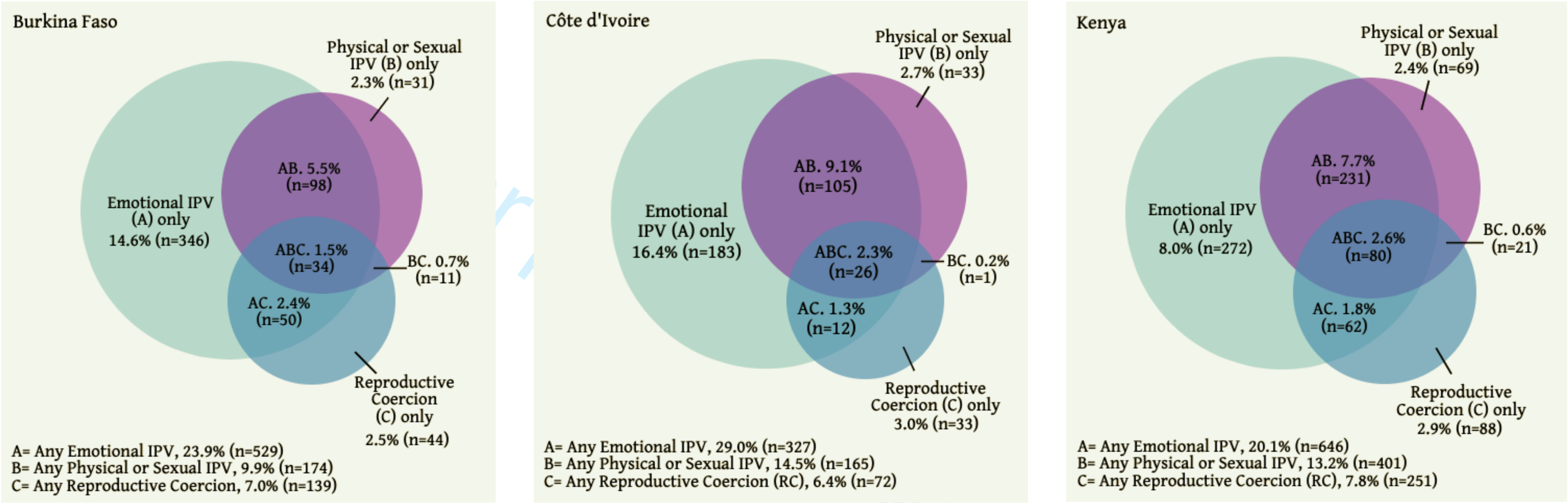

STROBE Statement—Checklist of items that should be included in reports of ***cross-sectional studies***

	Item No	Recommendation	Page No
**Title and abstract**	1	(*a*) Indicate the study's design with a commonly used term in the title or the abstract	1
		(*b*) Provide in the abstract an informative and balanced summary of what was done and what was found	2
**Introduction**			
Background/rationale	2	Explain the scientific background and rationale for the investigation being reported	4
Objectives	3	State specific objectives, including any prespecified hypotheses	4
**Methods**			
Study design	4	Present key elements of study design early in the paper	
Setting	5	Describe the setting, locations, and relevant dates, including periods of recruitment, exposure, follow-up, and data collection	5
Participants	6	(*a*) Give the eligibility criteria, and the sources and methods of selection of participants	5
Variables	7	Clearly define all outcomes, exposures, predictors, potential confounders, and effect modifiers. Give diagnostic criteria, if applicable	6
Data sources/ measurement	8*	For each variable of interest, give sources of data and details of methods of assessment (measurement). Describe comparability of assessment methods if there is more than one group	*6*
Bias	9	Describe any efforts to address potential sources of bias	8
Study size	10	Explain how the study size was arrived at	5-6
Quantitative variables	11	Explain how quantitative variables were handled in the analyses. If applicable, describe which groupings were chosen and why	6
Statistical methods	12	(*a*) Describe all statistical methods, including those used to control for confounding	6
		(*b*) Describe any methods used to examine subgroups and interactions	NA
		(*c*) Explain how missing data were addressed	6
		(*d*) If applicable, describe analytical methods taking account of sampling strategy	NA
		(*e*) Describe any sensitivity analyses	NA
**Results**			
Participants	13*	(a) Report numbers of individuals at each stage of study—eg numbers potentially eligible, examined for eligibility, confirmed eligible, included in the study, completing follow-up, and analysed	5-6
		(b) Give reasons for non-participation at each stage	5-6
		(c) Consider use of a flow diagram	NA
Descriptive data	14*	(a) Give characteristics of study participants (eg demographic, clinical, social) and information on exposures and potential confounders	7
		(b) Indicate number of participants with missing data for each variable of interest	6
Outcome data	15*	Report numbers of outcome events or summary measures	7
Main results	16	(*a*) Give unadjusted estimates and, if applicable, confounder-adjusted estimates and their precision (eg, 95% confidence interval). Make clear which confounders were adjusted for and why they were included	7

For peer review only

		(*b*) Report category boundaries when continuous variables were categorized	NA
		(*c*) If relevant, consider translating estimates of relative risk into absolute risk for a meaningful time period	NA
Other analyses	17	Report other analyses done—eg analyses of subgroups and interactions, and sensitivity analyses	7
**Discussion**			
Key results	18	Summarise key results with reference to study objectives	7-8
Limitations	19	Discuss limitations of the study, taking into account sources of potential bias or imprecision. Discuss both direction and magnitude of any potential bias	8
Interpretation	20	Give a cautious overall interpretation of results considering objectives, limitations, multiplicity of analyses, results from similar studies, and other relevant evidence	8
Generalisability	21	Discuss the generalisability (external validity) of the study results	8
**Other information**			
Funding	22	Give the source of funding and the role of the funders for the present study and, if applicable, for the original study on which the present article is based	In system

*Give information separately for exposed and unexposed groups.

Note: An Explanation and Elaboration article discusses each checklist item and gives methodological background and published examples of transparent reporting. The STROBE checklist is best used in conjunction with this article (freely available on the Web sites of PLoS Medicine at http://www.plosmedicine.org/, Annals of Internal Medicine at http://www.annals.org/, and Epidemiology at http://www.epidem.com/). Information on the STROBE Initiative is available at www.strobe-statement.org.

For peer review only

For peer review only - http://bmjopen.bmj.com/site/about/guidelines.xhtml

BMJ Open

The intersection of reproductive coercion and intimate partner violence: cross-sectional influences on women's contraceptive use in Burkina Faso, Côte d'Ivoire, and Kenya

Journal:	*BMJ Open*
Manuscript ID	bmjopen-2022-065697.R1
Article Type:	Original research
Date Submitted by the Author:	28-Nov-2022

Complete List of Authors:	Wood, Shannon; Johns Hopkins University Bloomberg School of Public Health, Department of Population, Family and Reproductive Health; Johns Hopkins University Bloomberg School of Public Health, Bill & Melinda Gates Institute for Population and Reproductive Health<br>Thomas, Haley; Johns Hopkins University Bloomberg School of Public Health, Department of Population, Family and Reproductive Health<br>Thiongo, Mary; International Centre for Reproductive Health Kenya<br>Guiella, Georges; Institut Supérieur des Sciences de la Population<br>Bazié, Fiacre; Institut Supérieur des Sciences de la Population<br>Onadja, Yentéma; Institut Supérieur des Sciences de la Population<br>Mosso, Rosine; Ecole Nationale Superieure de Statistique et d'Economie Appliquee<br>Fassassi, Raimi; Ecole Nationale Superieure de Statistique et d'Economie Appliquee<br>Gichangi, Peter; International Centre for Reproductive Health Kenya; Technical University of Mombasa<br>Decker, Michele; Johns Hopkins University Bloomberg School of Public Health, Population, Family and Reproductive Health

**Primary Subject Heading**:	Global health
Secondary Subject Heading:	Obstetrics and gynaecology
Keywords:	REPRODUCTIVE MEDICINE, PUBLIC HEALTH, GYNAECOLOGY

SCHOLARONE™
Manuscripts

I, the Submitting Author has the right to grant and does grant on behalf of all authors of the Work (as defined in the below author licence), an exclusive licence and/or a non-exclusive licence for contributions from authors who are: i) UK Crown employees; ii) where BMJ has agreed a CC-BY licence shall apply, and/or iii) in accordance with the terms applicable for US Federal Government officers or employees acting as part of their official duties; on a worldwide, perpetual, irrevocable, royalty-free basis to BMJ Publishing Group Ltd ("BMJ") its licensees and where the relevant Journal is co-owned by BMJ to the co-owners of the Journal, to publish the Work in this journal and any other BMJ products and to exploit all rights, as set out in our licence.

The Submitting Author accepts and understands that any supply made under these terms is made by BMJ to the Submitting Author unless you are acting as an employee on behalf of your employer or a postgraduate student of an affiliated institution which is paying any applicable article publishing charge ("APC") for Open Access articles. Where the Submitting Author wishes to make the Work available on an Open Access basis (and intends to pay the relevant APC), the terms of reuse of such Open Access shall be governed by a Creative Commons licence – details of these licences and which Creative Commons licence will apply to this Work are set out in our licence referred to above.

Other than as permitted in any relevant BMJ Author's Self Archiving Policies, I confirm this Work has not been accepted for publication elsewhere, is not being considered for publication elsewhere and does not duplicate material already published. I confirm all authors consent to publication of this Work and authorise the granting of this licence.

For peer review only

The intersection of reproductive coercion and intimate partner violence: cross-sectional influences on women's contraceptive use in Burkina Faso, Côte d'Ivoire, and Kenya

Shannon N. Wood[1,2]
Haley L. Thomas[1]
Mary Thiongo[3]
Georges Guiella[4]
Fiacre Bazié [4]
Yentéma Onadja[4]
Rosine Mosso[5]
Raimi Fassassi[5]
Peter Gichangi[3,6,7]
Michele R. Decker[1,2,8]

[1] Department of Population, Family and Reproductive Health, Johns Hopkins Bloomberg School of Public Health, Baltimore, USA.
[2] Bill & Melinda Gates Institute for Population and Reproductive Health, Department of Population, Family and Reproductive Health, Johns Hopkins Bloomberg School of Public Health, Baltimore, USA.
[3] International Centre for Reproductive Health-Kenya, Nairobi, Kenya.
[4] Institut Supérieur des Sciences de la Population (ISSP/Université Joseph Ki-Zerbo), Ouagadougou, Burkina Faso
[5] Ecole Nationale Superieure de Statistique et d'Economie Appliquee (ENSEA), Abidjan, Cote d'Ivoire.
[6] Technical University of Mombasa, Mombasa, Kenya.
[7] Department of Public Health and Primary Care, Faculty of Medicine and Health Sciences, Ghent University, Belgium
[8] Johns Hopkins School of Nursing, Baltimore, USA.

Corresponding author
Shannon N. Wood
615 N Wolfe St, E4009
Baltimore, MD 21205
swood@jhu.edu

Number of references: 31
Abstract word count: 312
Total word count: 3042

For peer review only

Abstract

Objectives: Among nationally representative cross-sections of women in need of contraception from Burkina Faso, Côte d'Ivoire, and Kenya, we aimed to: 1) examine the intersection of past-year physical/sexual intimate partner violence (IPV), emotional IPV, and reproductive coercion (RC); 2) assess the impact of physical/sexual IPV, emotional IPV, and RC on women's contraceptive use outcomes, including current contraceptive use, method type, and covert use.

Methods: The present analysis uses cross-sectional female data collected in Burkina Faso (December 2020-March 2021), Côte d'Ivoire (October-November 2021) and Kenya (November-December 2020). Analytic samples were restricted to partnered women with contraceptive need who completed a violence module (Burkina Faso n=1,863; Côte d'Ivoire n=1105; Kenya n=3390). The exposures of interest—past-year emotional IPV, physical/sexual IPV, and RC—were assessed using abridged versions of the Revised Conflict and Tactics Scale (CTS-2) and Reproductive Coercion Scale, respectively. Outcomes of interest included current contraceptive use, contraceptive method type (female controlled vs. male compliant), and covert contraceptive use, and utilized standard assessments. By site, prevalence and overlap of IPV and RC experiences were calculated, and bivariate and multivariable logistic regression used to examine outcomes by violence exposure.

Results: Across sites, 6.4% (Côte d'Ivoire) to 7.8% (Kenya) of women in need of contraception experienced RC; approximately one-third to one-half of women experiencing RC reported no other violence forms (31.7% in Burkina Faso to 45.8% in Côte d'Ivoire), whereas physical/sexual IPV largely occurred with emotional IPV. In multivariable models, RC was consistently associated with covert use (Burkina Faso: aOR=2.84 [95% CI=1.21-6.64]; Côte d'Ivoire: aOR =4.45 [95% CI=1.76-11.25]; Kenya: aOR=5.77 [95% CI=3.51-9.46]). Some IPV in some settings was also associated with covert use (emotional IPV, Burkina Faso: aOR=2.99 [95% CI=1.56-5.74]; physical/sexual, Kenya: aOR=2.35 [95% CI=1.33-4.17]).

Conclusions: Across settings, covert use is a critical strategy for women experiencing RC. Country policies must recognize RC as a unique form of violence with profound implications for women's reproductive health.

For peer review only - http://bmjopen.bmj.com/site/about/guidelines.xhtml

Strengths and Limitations

- Concurrent measurement of RC and IPV within three national samples (Burkina Faso, Cote d'Ivoire and Kenya) to afforded examination of concurrent violence experiences.
- Given inclusion of comprehensive measures, there was a unique opportunity to examine the interplay of violence experiences and their impact on three contraceptive use outcomes (current contraceptive use, contraceptive method type, covert use).
- Findings are cross-sectional, limiting conclusions surrounding temporality of associations.
- While RC measurement utilizes the pregnancy coercion sub-scale from the RC Scale, the most comprehensive measure for RC to date, RC items do not account for a wide range of RC behaviors, and therefore may underestimate the true burden of RC in these contexts.

For peer review only

For peer review only - http://bmjopen.bmj.com/site/about/guidelines.xhtml

Introduction

Intimate partner violence (IPV) is pervasive globally—recent evidence highlights that 27% of women are affected by physical or sexual IPV.[1] Partners can further harm women's health and livelihoods through psychological and economic abuse and coercive control.[2,3] Reproductive coercion (RC), or partner control over contraceptive use and reproductive health trajectories,[4,5] was initially conceptualized as a unique form of violence that could occur in tandem with physical or sexual IPV.[6,7] RC,[6,7] like IPV,[8] has a negative impact on women's reproductive health trajectories and has been linked to decreased use of effective contraception and increased risk of unintended pregnancy.

Current evidence examining the reproductive health impact of these leading forms of violence has been concentrated within high-income contexts, namely, the United States[6,7] and Australia.[9] Recent literature, however, highlights that RC is also common in sub-Saharan Africa, though behaviors, intentions, and motivations may differ in comparison to high-income contexts.[10–13] Specifically, partners may enact a broader range of RC behaviors with the means of asserting power over reproductive decision-making, including seeking to prevent pregnancy against a woman's wishes via behaviors such as forced contraceptive use.[10]

Understanding the impact of abusive partner dynamics on women's reproductive health in sub-Saharan Africa is critical given that sub-Saharan African women experience disproportionate adverse health burdens; specifically, they have the highest global rates of unintended pregnancy,[14] maternal mortality,[15] and unsafe abortion.[16] Multi-country studies have sought to understand the impact of IPV on contraceptive use with inconclusive findings.[17] Studies have reported increased contraceptive and covert use among IPV survivors,[12] whereas others have indicated decreased contraceptive use.[18] Moreover, such studies have largely failed to disentangle the impact of RC and emotional abuse (i.e. manipulation, intimidation, verbal abuse, etc.) on women's reproductive health outcomes, though women may be pressured or shamed when seeking to delay or limit childbearing; as such, these forms of violence may significantly impact contraceptive use and continuation.[11,13,17]

Inclusion of comprehensive RC measures within recent population-based surveys allows for the examination of the interplay of violence experiences and their impact on contraceptive use outcomes. To date, analyses examining linkages between unique forms of violence and contraceptive use have been limited given lack of RC and IPV measures concurrently included in national surveys. In three national samples across East and West Africa, this study aimed to: 1) examine the intersection of physical/sexual IPV, emotional IPV, and RC and 2) assess the impact of IPV and RC on women's contraceptive use outcomes (current contraceptive use, method type, and covert use).

Methods

Study Contexts

In 2019, Burkina Faso, Côte d'Ivoire, and Kenya ranked similarly on the United Nations Development Program (UNDP) Gender Inequality Index (Burkina Faso 0.594, rank 147 out of 162;[19] Côte d'Ivoire 0.638, rank 153;[20] Kenya 0.518, rank 126).[21] All three countries have ratified

For peer review only - http://bmjopen.bmj.com/site/about/guidelines.xhtml

the Convention on the Elimination of all Forms of Discrimination Against Women (CEDAW) and have taken steps to protect women from gender-based violence (GBV),[22] however, RC has not been formally included as form of violence within national policies.

Overview of PMA and GBV Module

Analyses utilize data from Performance Monitoring for Action (PMA), which administers annual population-based panel and cross-sectional questionnaires at the household, female, and facility levels using a multi-stage cluster design allowing for nationally and regionally representative estimates. Additional details are available at pmadata.org.

This analysis uses cross-sectional female data collected in Burkina Faso (December 2020-March 2021), Côte d'Ivoire (October-November 2021) and Kenya (November-December 2020). Sites were selected for the present analysis due to implementation of the PMA GBV module, which allowed for examination of both IPV and RC experiences.

All female household members ages 15-49 were eligible for the PMA female survey. In accordance with ethical best practices,[23,24] only one female per household was eligible for the GBV module. If there was more than one eligible female per household, one woman per household was randomly selected.

Training and Ethical Protections

Data were collected by trained resident enumerators (REs) using mobile phones equipped with Open Data Kit software; privacy checks were built into the survey. Prior to implementation, all REs completed a GBV-specific training, which emphasized maintaining confidentiality and privacy, asking non-judgmental questions, monitoring for emotional upset (i.e., crying, agitation, restlessness), and referring women to support services. In line with best practices,[23,24] all participants, regardless of violence disclosure or selection into the module, received a referral sheet for GBV and health resources.

All participants provided oral consent to participate. Approval was received by ethical review committees at Johns Hopkins School of Public Health, Comite D'Ethique Pour La Recherche en Sante, Ministere de la Recherche Scientifique et de L'Innovation, Ministere de la Sante in Burkina Faso, Comité National d'Ethique des Sciences de la Vie et de la Santé (CNESVS) in Côte d'Ivoire, and Kenyatta National Hospital-University of Nairobi Ethics and Research Committee College of Health Sciences in Kenya.

Patient and Public Involvement

The GBV module was included within select PMA surveys at the request of the Ministries of Health. Results are disseminated to in-country stakeholders at national and local events.

Analytic Samples

One woman per household was randomly selected to complete the GBV module (n=4,289 Burkina Faso; n=2,752 Côte d'Ivoire; n=6,870 Kenya); participants did not complete the GBV module if there were privacy concerns (n=60 Burkina Faso; n=88 Côte d'Ivoire; n=119 Kenya). IPV and RC items were only administered to married or partnered women for a final sample of 3,048 women in Burkina Faso, 1,852 women in Côte d'Ivoire, and 4,355 women in Kenya.

For peer review only - http://bmjopen.bmj.com/site/about/guidelines.xhtml

The analytic sample was further restricted to women in need of contraception, defined as being sexually active in the last 12 months, wanting to wait more than 12 months to have another child or not wanting any more children, not currently pregnant, and fecund, for a final sample of n=1,863 Burkina Faso, n=1,105 Côte d'Ivoire, n=3,390 Kenya. Analytic samples float to accommodate small amounts of missing data (<1%).

Measures

Three independent variables classified past-year experiences of violence perpetrated by a husband/partner (emotional IPV, physical/sexual IPV, and RC) via behavioral assessment. Past-year IPV was measured via 5-item abbreviated version of the Revised Conflict and Tactics Scale (CTS-2).[25] Specifically, emotional IPV was assessed via affirmative response to a single behavior (In the past 12 months has your husband/partner insulted you, yelled at you, screamed or made humiliating remarks). Physical/sexual IPV was assessed via affirmative response to any of the following items: in the past 12 months has your husband/partner slapped, hit, or physically hurt you; threatened with a weapon or attempted to strangle or kill you; pressured or insisted on having sex when you did not want to (without physical force); physically forced you to have sex when you did not want to. Past-year RC was measured via 6-items from the pregnancy coercion sub-scale of the Reproductive Coercion Scale[26] and previously adapted to the sub-Saharan African context,[12]and included the following items: in the past 12 months has your husband/partner mistreated you for wanting to use family planning; forced or pressured you to become pregnant; said he would leave if you did not get pregnant; said he would have a baby with someone else if you did not get pregnant; took away your family planning or prevented you from going to the clinic for family planning; hurt you physically because you did not get pregnant. The condom-manipulation sub-scale of the RC Scale was not included given that condoms are more likely to be used in these settings for preventing HIV/STIs than pregnancy.

Three dichotomous dependent contraceptive use variables (current contraceptive use, method classification [female-controlled or male compliant] and covert contraceptive use) were examined using standard assessments.[27] Current contraceptive use was determined from a single no/yes survey item. Method mix classification (binary—male compliant/female-controlled) was generated based on level of partner involvement needed in the reported contraceptive method. Specifically, current contraceptive users were asked to identify all contraceptive methods being used; the respondent's most effective method was then categorized as either a male compliant method or a female-controlled method, with female-controlled methods being those that women could use without partner involvement (female sterilization, implant, IUD, injectables, pills, EC, LAM), and male-compliant methods being those requiring men to actively use or accept women's use (male sterilization, male condom, female condom, diaphragm, foam/jelly, standard days/cycle beads, rhythm, withdrawal, other traditional). Direct covert use assessment was utilized to determine covert contraceptive use among current users,[28] where each contraceptive user was asked the following question: "does your husband/partner know that you are using *method reported*?" (response categories: no/yes).

Several sociodemographic and relationship characteristics were explored as potential confounders between violence and contraceptive use outcomes using standard assessments,[27] including

For peer review only - http://bmjopen.bmj.com/site/about/guidelines.xhtml

residence (urban/rural); parity (0, 1-2, 3+), marital status (married/living with a partner), polygyny (yes/no), and employment (yes/no).

Statistical Analysis

All analyses were stratified by site. Sociodemographic, relationship, and sexual and reproductive health characteristics were examined overall and among those with contraceptive need. The prevalence of past-year RC was calculated overall and by item; prevalence of past-year IPV was calculated overall and by IPV sub-type (emotional, physical/sexual). Overlapping experiences of emotional IPV, physical/sexual IPV, and RC were examined via Venn diagrams. Bivariate and multivariable logistic regression models were used to separately examine each contraceptive outcome by experience of violence; specifically, three models were utilized: 1) bivariate; 2) adjustment for sociodemographic and characteristics only; 3) adjustment for sociodemographic and relationship characteristics and other types of violence. Sociodemographic and relationship characteristics were examined for multi-collinearity, and ultimately, the third model was retained given <10% difference in effect estimates between models. All analyses were conducted in Stata version 16 (College Station, TX) and weighted to account for survey design.

Results

In Burkina Faso and Kenya, most women lived within rural areas, whereas in Côte d'Ivoire, residence was split between urban and rural localities (Table 1). Approximately 90% of partnered Burkinabe and Kenyan women were married compared to large proportions of women from Côte d'Ivoire lived with a partner outside of marriage (39.4%). Polygynous unions were highest in Burkina Faso (28.2%) and lowest in Kenya (11.4%). More than three-quarters of women in need of contraception were currently using contraception in Kenya (77.6%), whereas this proportion was substantially lower in Burkina Faso (52.6%) and Côte d'Ivoire (49.2%). In Kenya and Côte d'Ivoire, short-acting hormonal methods were the most used contraceptive methods (48.4% and 49.9%, respectively); in Burkina Faso, higher proportions of women used LARCs (45.5%). Approximately 11% of contraceptive users in Burkina Faso and Côte d'Ivoire used covertly; this proportion was slightly lower in Kenya (7.6%).

Table 1. Demographic Characteristics of Married/Partnered Women Selected for GBV Module, by Country

	Burkina Faso		Côte d'Ivoire		Kenya	
	Married/ Partnered women (n=3048)	FP need (n=1863)	Married/ Partnered women (n=1852)	FP need (n=1105)	Married/ Partnered women (n=4355)	FP need (n=3390)
	%*					
**Sociodemographic**						
Residence						
Urban	19.6	20.2	48.5	47.7	30.6	30.8
Rural	80.4	79.9	51.5	52.3	69.4	69.2
Household Wealth Tertile						
Lowest	35.1	37.4	39.1	39.6	37.0	36.7

Middle	33.8	32.5	31.0	31.3	33.6	33.8
Highest	31.1	30.1	29.9	29.1	29.5	29.5
Household composition						
Does not live with extended family	63.7	64.0	57.7	59.1	76.1	76.3
Lives with extended family	36.3	36.0	42.3	41.0	23.9	23.7
Age						
15-19	7.5	7.6	5.3	5.5	2.1	1.8
20-29	42.0	43.3	36.1	38.8	38.7	37.9
30-39	34.0	32.9	40.9	41.6	39.0	40.6
40-49	16.4	16.2	17.8	14.1	20.3	19.7
Education						
None	67.0	67.6	52.6	49.5	4.8	4.1
Primary	18.5	18.2	26.6	27.1	51.4	52.0
Secondary or Higher	14.5	14.2	20.7	23.4	43.9	43.9
Parity						
0	4.7	0.8	6.4	1.6	3.7	1.4
1-2	33.2	33.8	35.3	33.3	37.9	36.8
3+	62.2	65.4	58.3	65.2	58.5	61.8
**Relationship Dyad**						
Marital status						
Married	90.9	92.4	64.2	60.6	89.5	89.6
Living with partner	9.1	7.6	35.9	39.4	10.5	10.4
Polygamous union	30.5	28.2	18.4	16.6	12.1	11.4
Age at marriage						
≦15	5.3	4.9	11.9	11.8	7.0	7.1
>15 & <18	47.0	46.9	29.1	31.0	22.6	22.0
≧18	47.8	48.2	59.0	57.2	70.4	70.9
Partner education						
None	62.5	63.4	40.3	39.1	3.9	3.4
Primary	21.2	21.0	24.0	22.7	44.7	45.6
Secondary or Higher	16.3	15.6	35.7	38.2	51.4	51.0
**Finance**						
Employed	29.8	30.3	47.1	47.8	48.0	49.3
Has savings	15.3	15.4	8.7	8.7	48.0	48.1
Level of financial knowledge						
Not at all or not very	92.9	92.8	63.7	63.7	21.8	21.7
Somewhat or very	7.1	7.2	36.3	36.3	78.2	78.3
Economically reliant on partner for basic needs	51.8	52.4	56.2	56.4	61.9	61.4
**SRH**						
Currently pregnant	10.4	--	10.3	--	8.1	--
Feeling when found out about pregnancy[1]						
Very/sort of happy	74.5	--	67.2	--	66.9	--

For peer review only - http://bmjopen.bmj.com/site/about/guidelines.xhtml

Mixed happy and unhappy	1.8	--	7.3	--	12.9	--
Sort of/very unhappy	23.7	--	25.6	--	20.2	--
Currently using any contraceptives	36.0	52.6	32.8	49.2	66.1	77.6
Method mix, among users[2]						
Male Compliant	18.5	18.6	30.5	29.8	10.6	10.7
Female-controlled (LARC) †	45.7	45.5	19.8	19.5	41.5	39.4
Female-controlled (short-acting)	35.8	35.9	49.7	50.7	47.9	50.0
Covert contraceptive use	12.5	11.1	12.0	11.3	7.9	7.6
Feeling if you got pregnant now[3]						
Very/sort of happy	44.3	33.7	50.4	34.8	37.3	32.5
Mixed happy and unhappy	4.9	5.9	8.2	10.8	16.0	17.9
Sort of/very unhappy	50.8	60.4	41.4	54.4	46.6	49.7

[1] Among women who are currently pregnant

[2]

[3] Among women who are not currently pregnant

† Includes female sterilization (n=4 in Burkina Faso, n=0 in Côte d'Ivoire, n=102 in Kenya among the married/partnered samples)

Across sites, emotional IPV was the most prevalent type of violence experienced among women in need of contraception (20.1% Kenya, 23.9% Burkina Faso, 29.0% Côte d'Ivoire), followed by physical/sexual IPV (9.9% Burkina Faso, 13.2% Kenya, 14.5% Côte d'Ivoire) and RC (6.4% Côte d'Ivoire, 7.0% Burkina Faso, 7.8% Kenya; Table 2). Mistreatment for wanting to use family planning was the most common RC behavior (5.2% Burkina Faso, 4.4% Côte d'Ivoire, and 5.3% Kenya), whereas physical violence as a result of not getting pregnant was rarer (0.3% Burkina Faso, 0.4% Côte d'Ivoire, 1.2% Kenya).

Table 2. Prevalence of Past-Year Reproductive Coercion and Intimate Partner Violence among Married/Partnered Women with Need for Contraception, by Country

	Burkina Faso	Côte d'Ivoire	Kenya
	% weighted		
Individual RC Items			
In the past 12 months has your husband/partner…			
Mistreated you for wanting to use family planning	5.2	4.4	5.3
Forced or pressured you to become pregnant	2.2	3.2	4.3
Said he would leave if you did not get pregnant	1.3	1.6	1.9
Said he would have baby with someone else if you did not get pregnant	1.3	1.9	1.9

For peer review only - http://bmjopen.bmj.com/site/about/guidelines.xhtml

Took away your family planning or prevented you from going to clinic for family planning	1.6	0.9	2.0
Hurt you physically because you did not get pregnant	0.3	0.4	1.2
Any Experience of RC	7.0	6.4	7.8
IPV			
**Emotional IPV**	23.9	29.0	20.1
**Physical/sexual IPV**	9.9	14.5	13.2
Any Experience of IPV	26.9	32.1	23.1

Women in need of contraception experienced multiple types of violence simultaneously, with 10.1%, 12.9%, and 12.7% experiencing two of more types of violence in Burkina Faso, Côte d'Ivoire, and Kenya respectively (Figure 1). When exclusively examining reports of RC (n=139 Burkina Faso; n=72 Côte d'Ivoire; n=251 Kenya), approximately one-third to one-half of women reported RC experience in isolation (31.7% (44/139) Burkina Faso, 45.8% (33/72) Côte d'Ivoire, 35.1% (88/251) Kenya). In Burkina Faso, substantial portions women experiencing RC reported RC in tandem with emotional IPV (36.0% (50/139)), whereas in Côte d'Ivoire and Kenya, approximately one-third reported overlap with both emotional IPV and physical/sexual IPV (36.1% (26/72) and 31.9% (80/251), respectively).

RC was consistently associated with increased covert use of contraception across sites (Burkina Faso: aOR=2.84; 95% CI=1.21-6.64; Côte d'Ivoire: aOR=4.45; 95% CI=1.76-11.25; Kenya: aOR=5.77; 95% CI=3.51-9.46; Table 3). Emotional IPV was associated further with covert contraceptive use in Burkina Faso (aOR=2.99; 95% CI=1.56-5.74) and physical/sexual IPV with covert contraceptive use in Kenya (aOR=2.35; 95% CI=1.33-4.17). In Kenya, emotional IPV was associated with increased odds of current contraceptive use (aOR=1.44; CI=1.04-2.01), and in Burkina Faso, emotional IPV was marginally associated with current contraceptive use below the significance threshold (aOR=1.36; 95% CI=0.97-1.92; p=0.08). Also, in Burkina Faso, RC experience was associated with non-significant decreases in current contraceptive use (aOR=0.66; 95% CI=0.43-1.01; p=0.06). None of the violence forms displayed significant associations with method type.

For peer review only - http://bmjopen.bmj.com/site/about/guidelines.xhtml

Table 3. Logistic Regression between Past-Year RC and IPV and Contraceptive Use Outcomes Among Married/Partnered Women in Need of Contraception, per Country

**Burkina Faso**									
	Reproductive Coercion			Emotional IPV			Physical/ Sexual IPV		
	No RC (n=1719)	RC (n=139)	aOR[∝]	No IPV (n=1332)	IPV (n=529)	aOR[∝]	No IPV (n=1686)	IPV (n=174)	aOR[∝]
	%			%			%		
Current contraceptive use	53.2	46.2	0.66 (0.43, 1.01) ±	50.9	**58.2***	1.36 (0.97, 1.92) ±	52.6	52.7	0.91 (0.54, 1.54)
Method mix, among users									
Male compliant[1]	18.5	19.5	ref	19.5	15.9	ref	19.0	15.0	ref
Female-controlled[2]	81.5	80.6	0.81 (0.36, 1.86)	80.5	84.1	1.23 (0.72, 2.09)	81.1	85.0	1.14 (0.47, 2.73)
Covert contraceptive use	9.9	**29.3*****	**2.84 (1.21, 6.64)***	7.5	**20.9*****	**2.99 (1.56, 5.74)*****	10.3	17.9	0.83 (0.36, 1.91)
**Côte d'Ivoire**									
	RC			Emotional IPV			Physical/ Sexual IPV		
	No RC (n=1026)	RC (n=72)	aOR[∝]	No IPV (n=778)	IPV (n=327)	aOR[∝]	No IPV (n=940)	IPV (n=165)	aOR[∝]
	%			%			%		
Current contraceptive use	49.1	54.2	1.30 (0.72, 2.34)	48.1	52.0	1.15 (0.67, 1.97)	48.2	54.9%	1.25 (0.61, 2.56)
Method mix, among users									
Male compliant	30.7	19.3	ref	31.4	26.3	ref	30.8	24.5	ref
Female-controlled	69.3	80.7	--	68.7	73.7	1.20 (0.67, 2.14)	69.2	75.5	--

For peer review only

Covert contraceptive use	9.5	**35.0*****	**4.45 (1.76, 11.25)****	9.4	15.5	1.88 (0.87, 4.05)	10.9	13.0	0.59 (0.19, 1.86)
**Kenya**									
	RC			Emotional IPV			Physical/ Sexual IPV		
	No RC (n=3135)	RC (n=251)	aORœ	No IPV (n=2741)	IPV (n=646)	aORœ	No IPV (n=2986)	IPV (n=401)	aORœ
	%			%			%		
Current contraceptive use	77.9	74.2	0.84 (0.57, 1.23)	77.2	79.2	**1.44 (1.04, 2.01)***	77.8	76.3	0.82 (0.60, 1.13)
Method mix, among users									
Male compliant	10.7	12.1	ref	10.3	12.6	ref	10.6	11.5	ref
Female-controlled	89.4	87.9	0.97 (0.55, 1.69)	89.7	87.4	0.73 (0.50, 1.05)±	89.4	88.5	1.18 (0.67, 2.07)
Covert contraceptive use	5.6	**30.8*****	**5.77 (3.51, 9.46)*****	6.1	**13.2*****	0.8 (0.45, 1.42)	6.1	**17.5*****	**2.35 (1.33, 4.17)****

±<0.1; *<0.05; **<0.01; ***<0.001; bolding represents significance p<0.05

œaOR adjusted for other forms of violence, residence, education, parity, marital status, polygyny, employment

-- cells n<10 suppressed for regression

Male compliant: Male sterilization, male condom, female condom, diaphragm, foam/jelly, standard days/cycle beads, rhythm, withdrawal, other traditional; Female-controlled: Female sterilization, implant, IUD, injectables, pills, EC, LAM

For peer review only

Discussion

Leveraging large-scale, population-based samples, this analysis illuminates the prevalence of RC and overlap with physical/sexual and emotional IPV, and its impact on current and covert contraceptive use in Burkina Faso, Côte d'Ivoire, and Kenya. Data illustrate that for women experiencing RC who are in need of contraception, RC often occurred in isolation (31.7% in Burkina Faso to 45.8% in Côte d'Ivoire) or coupled with emotional IPV (16.7% in Côte d'Ivoire to 36.0% in Burkina Faso). Accordingly, only situating RC interventions within IPV programs and response services may overlook the needs of women who are experiencing this unique, yet detrimental, form of violence. Further, screening for physical or sexual IPV alone will fail to capture most RC experiences.

Notably, experience of past-year RC was consistently associated with increased covert use of contraception across sites (Burkina Faso: aOR=2.84; 95% CI=1.21-6.64; Côte d'Ivoire: aOR=4.45; 95% CI=1.76-11.25; Kenya: aOR=5.77; 95% CI=3.51-9.46), however, similar results were not observed for female-controlled method use. Covert use may prove a promising, yet risky safety strategy for women seeking to avert pregnancy and also experiencing RC.[12] Qualitative data with IPV and RC survivors in Nairobi indicated the cyclic covert use experiences of women—first electing to use contraception covertly given RC, then IPV or RC upon partner finding out about use, followed by a subsequent attempt to use a different contraceptive method covertly.[29] While providers may recommend female-controlled methods in light of partner disapproval, such as IUDs or implants, control of method use alone may not be enough to protect against unintended pregnancy when faced with RC. Instead, providers must be aware of RC and inquire about covert use preferences and potential RC experiences during contraceptive counseling to ensure method continuity and aversion of pregnancy without detriment to women's safety. Specifically, understanding previous experiences with side effects will be essential to ensure that women are able to use methods covertly. Further follow-up care will be necessary for women experiencing RC and covert use, as method switching may help minimize risk of partner suspicion.

Consistent with Demographic and Health Survey data, associations between physical/sexual IPV and contraceptive use were statistically insignificant,[17] however, emotional IPV was associated with significant increases in current contraceptive use in Kenya (aOR=1.44; 95% CI=1.04; p<0.05) and trend towards association in Burkina Faso (aOR=1.36; 95% CI=0.97-1.92; p=0.06). These results may signify important benchmarks on the abuse cycle and pathways to help-seeking—women wishing to avert pregnancy within violent relationships are acting on their reproductive preferences to utilize contraception. Further, they justify the inclusion of emotional abuse measures within large-scale surveys given its high prevalence and concurrence with RC.

Results must be considered in light of limitations. Foremost, findings are cross-sectional, limiting conclusions surrounding temporality of associations—longitudinal work is needed to disentangle whether women are using contraception because of experienced violence or whether the violence is incurred given contraceptive use. Further, while RC measures have improved since this form of violence of first conceptualized, abortion coercion and forced contraceptive use are still not components of the RC scale, limiting our ability to capture a more inclusive range of RC experiences. Our RC measures also did not account for condom use manipulation and thus may undercount the true burden of RC in these contexts.

Additionally, pregnant women were not included in the analytic sample, which could further underestimate RC prevalence as these women may be pregnant due to RC. However, including pregnant women in the sample would also lead to a systematic attenuation of the results between RC and contraceptive use given that these women are not in need of contraception. Women in dating partnerships were also excluded from the present analysis due to embedded survey skip logic, though evidence among urban adolescents and young women indicates high prevalence of RC;[30] further research and oversampling is needed to understand reproductive health needs for this high risk subpopulation. Social desirability biases and privacy concerns could further contribute to underreporting of experiences of violence, despite extensive training and privacy protocols aligned with best practices.[23,24]

Importantly, policymakers and family planning and violence service providers must recognize RC and its health impact to holistically support women's needs. Work to dismantle deep-rooted social norms that dictate spousal permission prior to contraceptive provision must be included in healthcare provider trainings on how to support women experiencing RC. Interventions such as ARCHES in Kenya[31] and have proven useful in decreasing RC through training family planning service providers to recognize RC as a prevalent form of violence that impacts women's reproductive health[31]—scale-up of such programs may help prevent recurrent violence. Integration of violence and reproductive health prevention and response services can further guarantee that women's needs are met regardless of type of abuse incurred. Standardization of recommendations can further ensure that women are able to enact safety strategies to protect their reproductive health while concurrently mitigating the impact of abuse. Though many countries have made recent notable steps towards GBV prevention and response, national policies do not currently recognize RC. Policies must not only name RC as a detriment to women's health, but also include practical rights-based solutions that ensure women's privacy in contraceptive decision-making and freedom from partner interference, such as universal, affordable, and judgement-free provision of covert contraceptive methods and emergency contraception, to ultimately counteract its reproductive health impact.

For peer review only - http://bmjopen.bmj.com/site/about/guidelines.xhtml

Ethics approval statement

Institutional Review Board approval was obtained at JHSPH (00009677), Comité d'Ethique pour la recherche en santé and the Ministère de la Santé et Ministère de l'Enseignement Supérieur, de la Recherche Scientifique et de l'Innovation (ISSP/DA/GG/062/2021), Comité d'Ethique de la Recherche Institut Pasteur de la Côte d'Ivoire (150-21/MSHP/CNESVS-km), and Kenya Medical Research Institute (KEMRI) Ethics Review Committee (KNH-ERC/Mod&SAE/314).

Contributorship statement

SNW: study design, oversight, analysis, initial draft. HLT: analysis, graphics, initial draft. MT, GG, FB, YO, RM, RF, PG: study design, oversight of data collection, ethical adherence, interpretation, and editing. MRD: study design, oversight, initial draft. All authors participated in writing and approving the final manuscript.

Competing interests

None declared.

Funding

This work was supported, in whole, by the Bill & Melinda Gates Foundation [009639]. Under the grant conditions of the Foundation, a Creative Commons Attribution 4.0 Generic License has already been assigned to the Author Accepted Manuscript version that might arise from this submission.

Data availability statement

Data are available upon request from pmadata.org.

For peer review only

References

1. Sardinha L, Maheu-Giroux M, Stöckl H, Meyer SR, García-Moreno C. Global, regional, and national prevalence estimates of physical or sexual, or both, intimate partner violence against women in 2018. *Lancet*. 2022;399(10327):803-813. doi:10.1016/S0140-6736(21)02664-7
2. Stylianou AM. Economic abuse within intimate partner violence: A review of the literature. *Violence Vict*. 2018;33(1):3-22. doi:10.1891/0886-6708.VV-D-16-00112
3. Heise L, Pallitto C, García-Moreno C, Clark CJ. Measuring psychological abuse by intimate partners: Constructing a cross-cultural indicator for the Sustainable Development Goals. *SSM - Popul Heal*. 2019;9(September 2018):1-6. doi:10.1016/j.ssmph.2019.100377
4. Silverman JG, Raj A. Intimate Partner Violence and Reproductive Coercion : Global Barriers to Women's Reproductive Control. *Plos Med*. 2014;11(9):9-12. doi:10.1371/journal.pmed.1001723
5. Grace KT, Anderson JC. Reproductive Coercion. *Trauma, Violence, Abus*. Published online 2016:1-20. doi:10.1177/1524838016663935
6. Miller E, Decker MR, McCauley HL, et al. Pregnancy coercion, intimate partner violence and unintended pregnancy. *Contraception*. 2010;81(4):316-322. doi:10.1016/j.contraception.2009.12.004
7. Miller E, Decker MR, McCauley HL, et al. A family planning clinic partner violence intervention to reduce risk associated with reproductive coercion. *Contraception*. 2011;83(3):274-280. doi:10.1016/j.contraception.2010.07.013
8. Bergmann JN, Stockman JK. How does intimate partner violence affect condom and oral contraceptive use in the United States ? A systematic review of the literature. *Contraception*. 2015;91:438-455.
9. Tarzia L, Srinivasan S, Marino J, Hegarty K. Exploring the gray areas between "stealthing" and reproductive coercion and abuse. *Women Heal*. 2020;60(10):1174-1184. doi:10.1080/03630242.2020.1804517
10. Wood SN, Kennedy SR, Akumu I, et al. Reproductive Coercion among Intimate Partner Violence Survivors in Nairobi. *Stud Fam Plann*. 2020;51(4):343-360.
11. Wood SN, Dozier J, Karp C, et al. Pregnancy Coercion, Correlates, and Associated Modern Contraceptive Use Within a Nationally Representative Sample of Ethiopian Women. In: *Population Association of America Annual Meeting*. ; 2021.
12. Silverman JG, Challa S, Boyce SC, Averbach S, Raj A. Associations of reproductive coercion and intimate partner violence with overt and covert family planning use among married adolescent girls in Niger. *EClinicalMedicine*. 2020;22(100359).
13. Boyce SC, Uysal J, Delong SM, et al. Women's and girls' experiences of reproductive coercion and opportunities for intervention in family planning clinics in Nairobi, Kenya: A qualitative study. *Reprod Health*. 2020;17(1):1-12. doi:10.1186/s12978-020-00942-7
14. Darroch JE, Singh S. Trends in contraceptive need and use in developing countries in 2003, 2008, and 2012: An analysis of national surveys. *Lancet*. 2013;381(9879):1756-1762. doi:10.1016/S0140-6736(13)60597-8
15. Alkema L, Chou D, Hogan D, et al. Global, regional, and national levels and trends in maternal mortality between 1990 and 2015, with scenario-based projections to 2030: a systematic analysis by the UN Maternal Mortality Estimation Inter-Agency Group. *Lancet*. 2016;387(10017):462-474. doi:10.1016/S0140-6736(15)00838-7
16. Tsui AO, McDonald-Mosley R, Burke AE. Family planning and the burden of unintended

For peer review only - http://bmjopen.bmj.com/site/about/guidelines.xhtml

pregnancies. *Epidemiol Rev*. 2010;32(1):152-174. doi:10.1093/epirev/mxq012
17. Macquarrie KLD, Mallick L, Kishor S. *DHS Analytical Studies 57: Intimate Partner Violence and Interruption to Contraceptive Use*.; 2016.
18. Maxwell L, H B, Ndyanabo A, et al. The impact of intimate partner violence on women's contraceptive use: Evidence from the Rakai Community Cohort Study in Rakai, Uganda. *Soc Sci Med*. 2018;209(April):25-32. doi:10.1016/j.socscimed.2018.04.050 LK - http://vb3lk7eb4t.search.serialssolutions.com?sid=EMBASE&issn=18735347&id=doi:10.1016%2Fj.socscimed.2018.04.050&atitle=The+impact+of+intimate+partner+violence+on+women%27s+contraceptive+use%3A+Evidence+from+the+Rakai+Community+Cohort+Study+in+Rakai%2C+Uganda&stitle=Soc.+Sci.+Med.&title=Social+Science+and+Medicine&volume=209&issue=&spage=25&epage=32&aulast=Maxwell&aufirst=Lauren&auinit=L.&aufull=Maxwell+L.&coden=SSMDE&isbn=&pages=25-32&date=2018&auinit1=L&aui
19. United Nations Development Programme. *Human Development Report 2020: The Next Frontier Human Development and the Anthropocene: Burkina Faso*.; 2020.
20. United Nations Development Programme. *Human Development Report 2020: The Next Frontier Human Development and the Anthropocene: Cote d'Ivoire*.; 2020.
21. United Nations Development Programme. *Human Development Report 2020: The Next Frontier Human Development and the Anthropocene: Kenya*.; 2020.
22. United Nations. Ratification Status for CEDAW-Convention on the Elimination of All Forms of Discrimination against Women. UN Treaty Body Database. Published 2022. Accessed April 4, 2022. https://tbinternet.ohchr.org/_layouts/15/TreatyBodyExternal/Treaty.aspx?Treaty=CEDAW&Lang=en
23. WHO. *Putting Women First: Ethical and Safety Recommendations for Research on Domestic Violence Against Women*.; 1999.
24. World Health Organization (WHO). *Ethical and Safety Recommendations for Intervention Research on Violence against Women*.; 2016.
25. Straus M, Hamby S, Boney-McCoy S, Sugarman D. The Revised Conflict Tactics Scales (CTS2). *J Fam Issues*. 1996;17(3):283-316.
26. McCauley HL, Silverman JG, Jones KA, et al. Psychometric properties and refinement of the Reproductive Coercion Scale. *Contraception*. 2017;95(3):292-298. doi:10.1016/j.contraception.2016.09.010
27. DHS. Demographic and Health Surveys: Model Woman's Questionnaire. 2016;(1):1-75.
28. Choiriyyah I, Becker S. Measuring Women's Covert Use of Modern Contraception in Cross-Sectional Surveys. *Stud Fam Plann*. 2018;49(2):143-157. doi:10.1111/sifp.12053
29. Wood SN, Kennedy SR, Akumu I, et al. Understanding the link between reproductive coercion and covert use of contraception as a safety strategy for women experiencing violence in Nairobi's urban informal settlements. *Violence Against Women Accept Press*. Published online 2022.
30. Decker MR, Wood SN, Byrne ME, et al. Gendered power dynamics and threats to sexual and reproductive autonomy among adolescent girls and young adult women: A cross-sectional survey in three urban settings. *PLoS One*. 2021;16(11 November):1-16. doi:10.1371/journal.pone.0257009
31. Uysal J, Carter N, Johns N, et al. Protocol for a matched-pair cluster control trial of ARCHES (Addressing Reproductive Coercion in Health Settings) among women and girls

For peer review only - http://bmjopen.bmj.com/site/about/guidelines.xhtml

1 seeking contraceptive services from community-based clinics in Nairobi, Kenya. *Reprod*
2 *Health*. 2020;17(1):1-14. doi:10.1186/s12978-020-00916-9

For peer review only

Figure 1. Venn Diagrams of IPV and Reproductive Coercion Experiences, Among Partnered Women with Need for Contraception, by Country

For peer review only

For peer review only - http://bmjopen.bmj.com/site/about/guidelines.xhtml

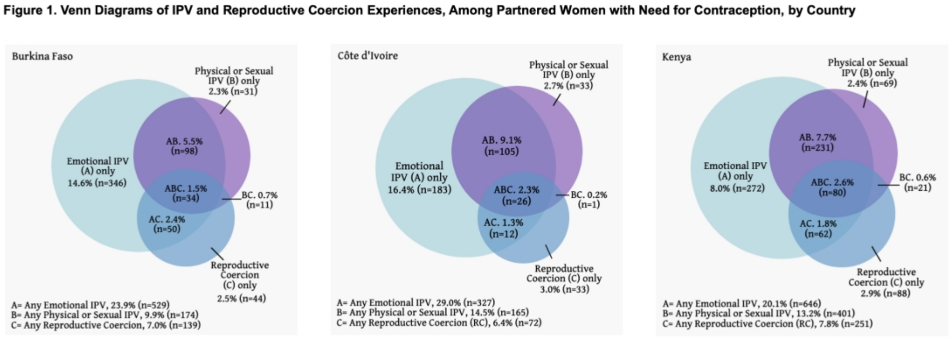

Venn Diagrams of IPV and Reproductive Coercion Experiences, Among Partnered Women with Need for Contraception, by Country

496x256mm (144 x 144 DPI)

STROBE Statement—Checklist of items that should be included in reports of ***cross-sectional studies***

	Item No	Recommendation	Page No
**Title and abstract**	1	(*a*) Indicate the study's design with a commonly used term in the title or the abstract	1
		(*b*) Provide in the abstract an informative and balanced summary of what was done and what was found	2
**Introduction**			
Background/rationale	2	Explain the scientific background and rationale for the investigation being reported	4
Objectives	3	State specific objectives, including any prespecified hypotheses	4
**Methods**			
Study design	4	Present key elements of study design early in the paper	
Setting	5	Describe the setting, locations, and relevant dates, including periods of recruitment, exposure, follow-up, and data collection	5
Participants	6	(*a*) Give the eligibility criteria, and the sources and methods of selection of participants	5
Variables	7	Clearly define all outcomes, exposures, predictors, potential confounders, and effect modifiers. Give diagnostic criteria, if applicable	6
Data sources/ measurement	8*	For each variable of interest, give sources of data and details of methods of assessment (measurement). Describe comparability of assessment methods if there is more than one group	*6*
Bias	9	Describe any efforts to address potential sources of bias	8
Study size	10	Explain how the study size was arrived at	5-6
Quantitative variables	11	Explain how quantitative variables were handled in the analyses. If applicable, describe which groupings were chosen and why	6
Statistical methods	12	(*a*) Describe all statistical methods, including those used to control for confounding	6
		(*b*) Describe any methods used to examine subgroups and interactions	NA
		(*c*) Explain how missing data were addressed	6
		(*d*) If applicable, describe analytical methods taking account of sampling strategy	NA
		(*e*) Describe any sensitivity analyses	NA
**Results**			
Participants	13*	(a) Report numbers of individuals at each stage of study—eg numbers potentially eligible, examined for eligibility, confirmed eligible, included in the study, completing follow-up, and analysed	5-6
		(b) Give reasons for non-participation at each stage	5-6
		(c) Consider use of a flow diagram	NA
Descriptive data	14*	(a) Give characteristics of study participants (eg demographic, clinical, social) and information on exposures and potential confounders	7
		(b) Indicate number of participants with missing data for each variable of interest	6
Outcome data	15*	Report numbers of outcome events or summary measures	7
Main results	16	(*a*) Give unadjusted estimates and, if applicable, confounder-adjusted estimates and their precision (eg, 95% confidence interval). Make clear which confounders were adjusted for and why they were included	7

For peer review only

		(*b*) Report category boundaries when continuous variables were categorized	NA
		(*c*) If relevant, consider translating estimates of relative risk into absolute risk for a meaningful time period	NA
Other analyses	17	Report other analyses done—eg analyses of subgroups and interactions, and sensitivity analyses	7
**Discussion**			
Key results	18	Summarise key results with reference to study objectives	7-8
Limitations	19	Discuss limitations of the study, taking into account sources of potential bias or imprecision. Discuss both direction and magnitude of any potential bias	8
Interpretation	20	Give a cautious overall interpretation of results considering objectives, limitations, multiplicity of analyses, results from similar studies, and other relevant evidence	8
Generalisability	21	Discuss the generalisability (external validity) of the study results	8
**Other information**			
Funding	22	Give the source of funding and the role of the funders for the present study and, if applicable, for the original study on which the present article is based	In system

*Give information separately for exposed and unexposed groups.

Note: An Explanation and Elaboration article discusses each checklist item and gives methodological background and published examples of transparent reporting. The STROBE checklist is best used in conjunction with this article (freely available on the Web sites of PLoS Medicine at http://www.plosmedicine.org/, Annals of Internal Medicine at http://www.annals.org/, and Epidemiology at http://www.epidem.com/). Information on the STROBE Initiative is available at www.strobe-statement.org.

For peer review only

For peer review only - http://bmjopen.bmj.com/site/about/guidelines.xhtml

BMJ Open

The intersection of reproductive coercion and intimate partner violence: cross-sectional influences on women's contraceptive use in Burkina Faso, Côte d'Ivoire, and Kenya

Journal:	*BMJ Open*
Manuscript ID	bmjopen-2022-065697.R2
Article Type:	Original research
Date Submitted by the Author:	10-Mar-2023
Complete List of Authors:	Wood, Shannon; Johns Hopkins University Bloomberg School of Public Health, Department of Population, Family and Reproductive Health; Johns Hopkins University Bloomberg School of Public Health, Bill & Melinda Gates Institute for Population and Reproductive Health<br>Thomas, Haley; Johns Hopkins University Bloomberg School of Public Health, Department of Population, Family and Reproductive Health<br>Thiongo, Mary; International Centre for Reproductive Health Kenya<br>Guiella, Georges; Institut Supérieur des Sciences de la Population<br>Bazié, Fiacre; Institut Supérieur des Sciences de la Population<br>Onadja, Yentéma; Institut Supérieur des Sciences de la Population<br>Mosso, Rosine; Ecole Nationale Superieure de Statistique et d'Economie Appliquee<br>Fassassi, Raimi; Ecole Nationale Superieure de Statistique et d'Economie Appliquee<br>Gichangi, Peter; International Centre for Reproductive Health Kenya; Technical University of Mombasa<br>Decker, Michele; Johns Hopkins University Bloomberg School of Public Health, Population, Family and Reproductive Health
**Primary Subject Heading**:	Global health
Secondary Subject Heading:	Obstetrics and gynaecology
Keywords:	REPRODUCTIVE MEDICINE, PUBLIC HEALTH, GYNAECOLOGY

SCHOLARONE™
Manuscripts

I, the Submitting Author has the right to grant and does grant on behalf of all authors of the Work (as defined in the below author licence), an exclusive licence and/or a non-exclusive licence for contributions from authors who are: i) UK Crown employees; ii) where BMJ has agreed a CC-BY licence shall apply, and/or iii) in accordance with the terms applicable for US Federal Government officers or employees acting as part of their official duties; on a worldwide, perpetual, irrevocable, royalty-free basis to BMJ Publishing Group Ltd ("BMJ") its licensees and where the relevant Journal is co-owned by BMJ to the co-owners of the Journal, to publish the Work in this journal and any other BMJ products and to exploit all rights, as set out in our licence.

The Submitting Author accepts and understands that any supply made under these terms is made by BMJ to the Submitting Author unless you are acting as an employee on behalf of your employer or a postgraduate student of an affiliated institution which is paying any applicable article publishing charge ("APC") for Open Access articles. Where the Submitting Author wishes to make the Work available on an Open Access basis (and intends to pay the relevant APC), the terms of reuse of such Open Access shall be governed by a Creative Commons licence – details of these licences and which Creative Commons licence will apply to this Work are set out in our licence referred to above.

Other than as permitted in any relevant BMJ Author's Self Archiving Policies, I confirm this Work has not been accepted for publication elsewhere, is not being considered for publication elsewhere and does not duplicate material already published. I confirm all authors consent to publication of this Work and authorise the granting of this licence.

For peer review only

The intersection of reproductive coercion and intimate partner violence: cross-sectional influences on women's contraceptive use in Burkina Faso, Côte d'Ivoire, and Kenya

Shannon N. Wood[1,2]
Haley L. Thomas[1]
Mary Thiongo[3]
Georges Guiella[4]
Fiacre Bazié [4]
Yentéma Onadja[4]
Rosine Mosso[5]
Raimi Fassassi[5]
Peter Gichangi[3,6,7]
Michele R. Decker[1,2,8]

[1] Department of Population, Family and Reproductive Health, Johns Hopkins Bloomberg School of Public Health, Baltimore, USA.
[2] Bill & Melinda Gates Institute for Population and Reproductive Health, Department of Population, Family and Reproductive Health, Johns Hopkins Bloomberg School of Public Health, Baltimore, USA.
[3] International Centre for Reproductive Health-Kenya, Nairobi, Kenya.
[4] Institut Supérieur des Sciences de la Population (ISSP/Université Joseph Ki-Zerbo), Ouagadougou, Burkina Faso
[5] Ecole Nationale Superieure de Statistique et d'Economie Appliquee (ENSEA), Abidjan, Cote d'Ivoire.
[6] Technical University of Mombasa, Mombasa, Kenya.
[7] Department of Public Health and Primary Care, Faculty of Medicine and Health Sciences, Ghent University, Belgium
[8] Johns Hopkins School of Nursing, Baltimore, USA.

Corresponding author
Shannon N. Wood
615 N Wolfe St, E4009
Baltimore, MD 21205
swood@jhu.edu

Number of references: 31
Abstract word count: 312
Total word count: 3042

For peer review only

Abstract

Objectives: Among nationally representative cross-sections of women in need of contraception from Burkina Faso, Côte d'Ivoire, and Kenya, we aimed to: 1) examine the intersection of past-year physical/sexual intimate partner violence (IPV), emotional IPV, and reproductive coercion (RC); 2) assess the impact of physical/sexual IPV, emotional IPV, and RC on women's contraceptive use outcomes, including current contraceptive use, method type, and covert use.

Methods: The present analysis uses cross-sectional female data collected in Burkina Faso (December 2020-March 2021), Côte d'Ivoire (October-November 2021) and Kenya (November-December 2020). Analytic samples were restricted to partnered women with contraceptive need who completed a violence module (Burkina Faso n=1,863; Côte d'Ivoire n=1105; Kenya n=3390). The exposures of interest—past-year emotional IPV, physical/sexual IPV, and RC—were assessed using abridged versions of the Revised Conflict and Tactics Scale (CTS-2) and Reproductive Coercion Scale, respectively. Outcomes of interest included current contraceptive use, contraceptive method type (female controlled vs. male compliant), and covert contraceptive use, and utilized standard assessments. By site, prevalence and overlap of IPV and RC experiences were calculated, and bivariate and multivariable logistic regression used to examine outcomes by violence exposure.

Results: Across sites, 6.4% (Côte d'Ivoire) to 7.8% (Kenya) of women in need of contraception experienced RC; approximately one-third to one-half of women experiencing RC reported no other violence forms (31.7% in Burkina Faso to 45.8% in Côte d'Ivoire), whereas physical/sexual IPV largely occurred with emotional IPV. In multivariable models, RC was consistently associated with covert use (Burkina Faso: aOR=2.84 [95% CI=1.21-6.64]; Côte d'Ivoire: aOR =4.45 [95% CI=1.76-11.25]; Kenya: aOR=5.77 [95% CI=3.51-9.46]). Some IPV in some settings was also associated with covert use (emotional IPV, Burkina Faso: aOR=2.99 [95% CI=1.56-5.74]; physical/sexual, Kenya: aOR=2.35 [95% CI=1.33-4.17]).

Conclusions: Across settings, covert use is a critical strategy for women experiencing RC. Country policies must recognize RC as a unique form of violence with profound implications for women's reproductive health.

For peer review only - http://bmjopen.bmj.com/site/about/guidelines.xhtml

Strengths and Limitations

- Concurrent measurement of RC and IPV within three national samples (Burkina Faso, Cote d'Ivoire and Kenya) afforded examination of concurrent violence experiences.
- Given inclusion of comprehensive measures, there was a unique opportunity to examine the interplay of violence experiences and their impact on three contraceptive use outcomes (current contraceptive use, contraceptive method type, covert use).
- Findings are cross-sectional, limiting conclusions surrounding temporality of associations.
- While RC measurement utilizes the pregnancy coercion sub-scale from the RC Scale, the most comprehensive measure for RC to date, RC items do not account for a wide range of RC behaviors, and therefore may underestimate the true burden of RC in these contexts.

For peer review only

For peer review only - http://bmjopen.bmj.com/site/about/guidelines.xhtml

Introduction

Intimate partner violence (IPV) is pervasive globally—recent evidence highlights that 27% of women are affected by physical or sexual IPV.[1] Partners can further harm women's health and livelihoods through psychological and economic abuse and coercive control.[2,3] Reproductive coercion (RC), or partner control over contraceptive use and reproductive health trajectories,[4,5] was initially conceptualized as a unique form of violence that could occur in tandem with physical or sexual IPV.[6,7] RC,[6,7] like IPV,[8] has a negative impact on women's reproductive health trajectories and has been linked to decreased use of effective contraception and increased risk of unintended pregnancy.

Current evidence examining the reproductive health impact of these leading forms of violence has been concentrated within high-income contexts, namely, the United States[6,7] and Australia.[9] Recent literature, however, highlights that RC is also common in sub-Saharan Africa, though behaviors, intentions, and motivations may differ in comparison to high-income contexts.[10–13] Specifically, partners may enact a broader range of RC behaviors with the means of asserting power over reproductive decision-making, including seeking to prevent pregnancy against a woman's wishes via behaviors such as forced contraceptive use.[10]

Understanding the impact of abusive partner dynamics on women's reproductive health in sub-Saharan Africa is critical given that sub-Saharan African women experience disproportionate adverse health burdens; specifically, they have the highest global rates of unintended pregnancy,[14] maternal mortality,[15] and unsafe abortion.[16] Multi-country studies have sought to understand the impact of IPV on contraceptive use with inconclusive findings.[17] Studies have reported increased contraceptive and covert use among IPV survivors,[12] whereas others have indicated decreased contraceptive use.[18] Moreover, such studies have largely failed to disentangle the impact of RC and emotional abuse (i.e. manipulation, intimidation, verbal abuse, etc.) on women's reproductive health outcomes, though women may be pressured or shamed when seeking to delay or limit childbearing; as such, these forms of violence may significantly impact contraceptive use and continuation.[11,13,17]

Inclusion of comprehensive RC measures within recent population-based surveys allows for the examination of the interplay of violence experiences and their impact on contraceptive use outcomes. To date, analyses examining linkages between unique forms of violence and contraceptive use have been limited given lack of RC and IPV measures concurrently included in national surveys. In three national samples across East and West Africa, this study aimed to: 1) examine the intersection of physical/sexual IPV, emotional IPV, and RC and 2) assess the impact of IPV and RC on women's contraceptive use outcomes (current contraceptive use, method type, and covert use).

Methods

Study Contexts

In 2019, Burkina Faso, Côte d'Ivoire, and Kenya ranked similarly on the United Nations Development Program (UNDP) Gender Inequality Index (Burkina Faso 0.594, rank 147 out of 162;[19] Côte d'Ivoire 0.638, rank 153;[20] Kenya 0.518, rank 126).[21] All three countries have ratified

For peer review only - http://bmjopen.bmj.com/site/about/guidelines.xhtml

the Convention on the Elimination of all Forms of Discrimination Against Women (CEDAW) and have taken steps to protect women from gender-based violence (GBV),[22] however, RC has not been formally included as form of violence within national policies.

Overview of PMA and GBV Module

Analyses utilize data from Performance Monitoring for Action (PMA), which administers annual population-based panel and cross-sectional questionnaires at the household, female, and facility levels using a multi-stage cluster design allowing for nationally and regionally representative estimates. Additional details are available at pmadata.org.

This analysis uses cross-sectional female data collected in Burkina Faso (December 2020-March 2021), Côte d'Ivoire (October-November 2021) and Kenya (November-December 2020). Sites were selected for the present analysis due to implementation of the PMA GBV module, which allowed for examination of both IPV and RC experiences.

All female household members ages 15-49 were eligible for the PMA female survey. In accordance with ethical best practices,[23,24] only one female per household was eligible for the GBV module. If there was more than one eligible female per household, one woman per household was randomly selected.

Training and Ethical Protections

Data were collected by trained resident enumerators (REs) using mobile phones equipped with Open Data Kit software; privacy checks were built into the survey. Prior to implementation, all REs completed a GBV-specific training, which emphasized maintaining confidentiality and privacy, asking non-judgmental questions, monitoring for emotional upset (i.e., crying, agitation, restlessness), and referring women to support services. In line with best practices,[23,24] all participants, regardless of violence disclosure or selection into the module, received a referral sheet for GBV and health resources.

All participants provided oral consent to participate. Approval was received by ethical review committees at Johns Hopkins School of Public Health, Comite D'Ethique Pour La Recherche en Sante, Ministere de la Recherche Scientifique et de L'Innovation, Ministere de la Sante in Burkina Faso, Comité National d'Ethique des Sciences de la Vie et de la Santé (CNESVS) in Côte d'Ivoire, and Kenyatta National Hospital-University of Nairobi Ethics and Research Committee College of Health Sciences in Kenya.

Patient and Public Involvement

The GBV module was included within select PMA surveys at the request of the Ministries of Health. Results are disseminated to in-country stakeholders at national and local events.

Analytic Samples

One woman per household was randomly selected to complete the GBV module (n=4,289 Burkina Faso; n=2,752 Côte d'Ivoire; n=6,870 Kenya); participants did not complete the GBV module if there were privacy concerns (n=60 Burkina Faso; n=88 Côte d'Ivoire; n=119 Kenya). IPV and RC items were only administered to married or partnered women for a final sample of 3,048 women in Burkina Faso, 1,852 women in Côte d'Ivoire, and 4,355 women in Kenya.

For peer review only - http://bmjopen.bmj.com/site/about/guidelines.xhtml

The analytic sample was further restricted to women in need of contraception, defined as being sexually active in the last 12 months, wanting to wait more than 12 months to have another child or not wanting any more children, not currently pregnant, and fecund, for a final sample of n=1,863 Burkina Faso, n=1,105 Côte d'Ivoire, n=3,390 Kenya. Analytic samples float to accommodate small amounts of missing data (<1%).

Measures

Three independent variables classified past-year experiences of violence perpetrated by a husband/partner (emotional IPV, physical/sexual IPV, and RC) via behavioral assessment. Past-year IPV was measured via 5-item abbreviated version of the Revised Conflict and Tactics Scale (CTS-2).[25] Specifically, emotional IPV was assessed via affirmative response to a single behavior (In the past 12 months has your husband/partner insulted you, yelled at you, screamed or made humiliating remarks). Physical/sexual IPV was assessed via affirmative response to any of the following items: in the past 12 months has your husband/partner slapped, hit, or physically hurt you; threatened with a weapon or attempted to strangle or kill you; pressured or insisted on having sex when you did not want to (without physical force); physically forced you to have sex when you did not want to. Past-year RC was measured via 6-items from the pregnancy coercion sub-scale of the RC Scale[26] and previously adapted to the sub-Saharan African context,[12]and included the following items: in the past 12 months has your husband/partner mistreated you for wanting to use family planning; forced or pressured you to become pregnant; said he would leave if you did not get pregnant; said he would have a baby with someone else if you did not get pregnant; took away your family planning or prevented you from going to the clinic for family planning; hurt you physically because you did not get pregnant. The condom-manipulation sub-scale of the RC Scale was not included given that condoms are more likely to be used in these settings for preventing HIV/STIs than pregnancy.

Three dichotomous dependent contraceptive use variables (current contraceptive use, method classification [female-controlled or male compliant] and covert contraceptive use) were examined using standard assessments.[27] Current contraceptive use was determined from a single no/yes survey item. Method mix classification (binary—male compliant/female-controlled) was generated based on level of partner involvement needed in the reported contraceptive method. Specifically, current contraceptive users were asked to identify all contraceptive methods being used; the respondent's most effective method was then categorized as either a male compliant method or a female-controlled method, with female-controlled methods being those that women could use without partner involvement (female sterilization, implant, intrauterine device (IUD), injectables, pills, emergency contraception (EC), lactational amenorrhea method (LAM)), and male-compliant methods being those requiring men to actively use or accept women's use, including cycle tracking methods given necessitation of compliance in timing of sexual activity (male sterilization, male condom, female condom, diaphragm, foam/jelly, standard days/cycle beads, rhythm, withdrawal, other traditional). Direct covert use assessment was utilized to determine covert contraceptive use among current users,[28] where each contraceptive user was asked the following question: "does your husband/partner know that you are using *method reported*?" (response categories: no/yes).

For peer review only - http://bmjopen.bmj.com/site/about/guidelines.xhtml

Several sociodemographic and relationship characteristics were explored as potential confounders between violence and contraceptive use outcomes using standard assessments,[27] including residence (urban/rural); parity (0, 1-2, 3+), marital status (married/living with a partner), polygyny (yes/no), and employment (yes/no).

Statistical Analysis

All analyses were stratified by site. Sociodemographic, relationship, and sexual and reproductive health characteristics were examined overall and among those with contraceptive need. The prevalence of past-year RC was calculated overall and by item; prevalence of past-year IPV was calculated overall and by IPV sub-type (emotional, physical/sexual). Overlapping experiences of emotional IPV, physical/sexual IPV, and RC were examined via Venn diagrams. Bivariate and multivariable logistic regression models were used to separately examine each contraceptive outcome by experience of violence; specifically, three models were utilized: 1) bivariate; 2) adjustment for sociodemographic and characteristics only; 3) adjustment for sociodemographic and relationship characteristics and other types of violence. Sociodemographic and relationship characteristics were examined for multi-collinearity, and ultimately, the third model was retained given <10% difference in effect estimates between models. All analyses were conducted in Stata version 16 (College Station, TX) and weighted to account for survey design.

Results

In Burkina Faso and Kenya, most women lived within rural areas, whereas in Côte d'Ivoire, residence was split between urban and rural localities (Table 1). Approximately 90% of partnered Burkinabe and Kenyan women were married compared to large proportions of women from Côte d'Ivoire lived with a partner outside of marriage (39.4%). Polygynous unions were highest in Burkina Faso (28.2%) and lowest in Kenya (11.4%). More than three-quarters of women in need of contraception were currently using contraception in Kenya (77.6%), whereas this proportion was substantially lower in Burkina Faso (52.6%) and Côte d'Ivoire (49.2%). In Kenya and Côte d'Ivoire, short-acting hormonal methods were the most used contraceptive methods (48.4% and 49.9%, respectively); in Burkina Faso, higher proportions of women used long-acting reversible contraceptives (LARCs) (45.5%). Approximately 11% of contraceptive users in Burkina Faso and Côte d'Ivoire used covertly; this proportion was slightly lower in Kenya (7.6%).

Table 1. Demographic Characteristics of Married/Partnered Women Selected for GBV Module, by Country

	Burkina Faso		Côte d'Ivoire		Kenya	
	Married/ Partnered women (n=3048)	FP need (n=1863)	Married/ Partnered women (n=1852)	FP need (n=1105)	Married/ Partnered women (n=4355)	FP need (n=3390)
	%*					
**Sociodemographic**						
Residence						
Urban	19.6	20.2	48.5	47.7	30.6	30.8
Rural	80.4	79.9	51.5	52.3	69.4	69.2

Household Wealth Tertile						
Lowest	35.1	37.4	39.1	39.6	37.0	36.7
Middle	33.8	32.5	31.0	31.3	33.6	33.8
Highest	31.1	30.1	29.9	29.1	29.5	29.5
Household composition						
Does not live with extended family	63.7	64.0	57.7	59.1	76.1	76.3
Lives with extended family	36.3	36.0	42.3	41.0	23.9	23.7
Age						
15-19	7.5	7.6	5.3	5.5	2.1	1.8
20-29	42.0	43.3	36.1	38.8	38.7	37.9
30-39	34.0	32.9	40.9	41.6	39.0	40.6
40-49	16.4	16.2	17.8	14.1	20.3	19.7
Education						
None	67.0	67.6	52.6	49.5	4.8	4.1
Primary	18.5	18.2	26.6	27.1	51.4	52.0
Secondary or Higher	14.5	14.2	20.7	23.4	43.9	43.9
Parity						
0	4.7	0.8	6.4	1.6	3.7	1.4
1-2	33.2	33.8	35.3	33.3	37.9	36.8
3+	62.2	65.4	58.3	65.2	58.5	61.8
**Relationship Dyad**						
Marital status						
Married	90.9	92.4	64.2	60.6	89.5	89.6
Living with partner	9.1	7.6	35.9	39.4	10.5	10.4
Polygamous union	30.5	28.2	18.4	16.6	12.1	11.4
Age at marriage						
≦15	5.3	4.9	11.9	11.8	7.0	7.1
>15 & <18	47.0	46.9	29.1	31.0	22.6	22.0
≧18	47.8	48.2	59.0	57.2	70.4	70.9
Partner education						
None	62.5	63.4	40.3	39.1	3.9	3.4
Primary	21.2	21.0	24.0	22.7	44.7	45.6
Secondary or Higher	16.3	15.6	35.7	38.2	51.4	51.0
**Finance**						
Employed	29.8	30.3	47.1	47.8	48.0	49.3
Has savings	15.3	15.4	8.7	8.7	48.0	48.1
Level of financial knowledge						
Not at all or not very	92.9	92.8	63.7	63.7	21.8	21.7
Somewhat or very	7.1	7.2	36.3	36.3	78.2	78.3
Economically reliant on partner for basic needs	51.8	52.4	56.2	56.4	61.9	61.4
**SRH**						
Currently pregnant	10.4	--	10.3	--	8.1	--
Feeling when found out about						

pregnancy[1]						
Very/sort of happy	74.5	--	67.2	--	66.9	--
Mixed happy and unhappy	1.8	--	7.3	--	12.9	--
Sort of/very unhappy	23.7	--	25.6	--	20.2	--
Currently using any contraceptives	36.0	52.6	32.8	49.2	66.1	77.6
Method mix, among users[2]						
Male Compliant	18.5	18.6	30.5	29.8	10.6	10.7
Female-controlled (LARC) †	45.7	45.5	19.8	19.5	41.5	39.4
Female-controlled (short-acting)	35.8	35.9	49.7	50.7	47.9	50.0
Covert contraceptive use	12.5	11.1	12.0	11.3	7.9	7.6
Feeling if you got pregnant now[3]						
Very/sort of happy	44.3	33.7	50.4	34.8	37.3	32.5
Mixed happy and unhappy	4.9	5.9	8.2	10.8	16.0	17.9
Sort of/very unhappy	50.8	60.4	41.4	54.4	46.6	49.7

[1] Among women who are currently pregnant

[2] Male compliant (male sterilization, male and female condoms, diaphragm, foam/jelly, standard days/cycle beads, rhythm, withdrawal, other traditional), female-controlled LARC (female sterilization, implant, IUD), and female-controlled short-acting (injectables, pills, EC, LAM).

[3] Among women who are not currently pregnant

† Includes female sterilization (n=4 in Burkina Faso, n=0 in Côte d'Ivoire, n=102 in Kenya among the married/partnered samples)

Across sites, emotional IPV was the most prevalent type of violence experienced among women in need of contraception (20.1% Kenya, 23.9% Burkina Faso, 29.0% Côte d'Ivoire), followed by physical/sexual IPV (9.9% Burkina Faso, 13.2% Kenya, 14.5% Côte d'Ivoire) and RC (6.4% Côte d'Ivoire, 7.0% Burkina Faso, 7.8% Kenya; Table 2). Mistreatment for wanting to use family planning was the most common RC behavior (5.2% Burkina Faso, 4.4% Côte d'Ivoire, and 5.3% Kenya), whereas physical violence as a result of not getting pregnant was rarer (0.3% Burkina Faso, 0.4% Côte d'Ivoire, 1.2% Kenya).

Table 2. Prevalence of Past-Year Reproductive Coercion and Intimate Partner Violence among Married/Partnered Women with Need for Contraception, by Country

	Burkina Faso	Côte d'Ivoire	Kenya
	% weighted		
Individual RC Items			
In the past 12 months has your husband/partner…			
Mistreated you for wanting to use family planning	5.2	4.4	5.3
Forced or pressured you to become pregnant	2.2	3.2	4.3
Said he would leave if you did not get pregnant	1.3	1.6	1.9
Said he would have baby with someone else if you did not get pregnant	1.3	1.9	1.9

Took away your family planning or prevented you from going to clinic for family planning	1.6	0.9	2.0
Hurt you physically because you did not get pregnant	0.3	0.4	1.2
Any Experience of RC	7.0	6.4	7.8
IPV			
**Emotional IPV**	23.9	29.0	20.1
**Physical/sexual IPV**	9.9	14.5	13.2
Any Experience of IPV	26.9	32.1	23.1

Women in need of contraception experienced multiple types of violence simultaneously, with 10.1%, 12.9%, and 12.7% experiencing two of more types of violence in Burkina Faso, Côte d'Ivoire, and Kenya respectively (Figure 1). When exclusively examining reports of RC (n=139 Burkina Faso; n=72 Côte d'Ivoire; n=251 Kenya), approximately one-third to one-half of women reported RC experience in isolation (31.7% (44/139) Burkina Faso, 45.8% (33/72) Côte d'Ivoire, 35.1% (88/251) Kenya). In Burkina Faso, substantial portions women experiencing RC reported RC in tandem with emotional IPV (36.0% (50/139)), whereas in Côte d'Ivoire and Kenya, approximately one-third reported overlap with both emotional IPV and physical/sexual IPV (36.1% (26/72) and 31.9% (80/251), respectively).

In bivariate analysis, RC was associated with covert contraceptive use in all three sites ($p<0.001$; Table 3). Emotional IPV was associated with increased use of contraception ($50.9\%_{\text{no emotional IPV}}$ vs. $58.2\%_{\text{emotional IPV}}$; $p<0.05$) in Burkina Faso only and increased covert contraceptive use in both Burkina Faso ($7.5\%_{\text{no emotional IPV}}$ vs. $20.9\%_{\text{emotional}}$ IPV; $p<0.001$) and Kenya ($6.1\%_{\text{no emotional IPV}}$ vs. $13.2\%_{\text{emotional}}$ IPV; $p<0.001$). Physical/sexual IPV was associated with covert contraceptive use in Kenya only ($6.1\%_{\text{no physical/sexual IPV}}$ vs. $17.5\%_{\text{physical/sexual IPV}}$; $p<0.001$).

For peer review only - http://bmjopen.bmj.com/site/about/guidelines.xhtml

Table 3. Bivariate Analysis between Past-Year RC and IPV and Contraceptive Use Outcomes Among Married/Partnered Women in Need of Contraception, per Country

	RC		Emotional IPV		Physical/ Sexual IPV	
**Burkina Faso**						
	No RC (n=1719)	RC (n=139)	No IPV (n=1332)	IPV (n=529)	No IPV (n=1686)	IPV (n=174)
	%		%		%	
Current contraceptive use	53.2	46.2	**50.9**	**58.2***	52.6	52.7
Method mix, among users						
Male compliant	18.5	19.5	19.5	15.9	19.0	15.0
Female controlled	81.5	80.6	80.5	84.1	81.1	85.0
Covert contraceptive use	**9.9**	**29.3*****	**7.5**	**20.9*****	10.3	17.9
**Cote d'Ivoire**						
	RC		Emotional IPV		Physical/ Sexual IPV	
	No RC (n=1026)	RC (n=72)	No IPV (n=778)	IPV (n=327)	No IPV (n=940)	IPV (n=165)
	%		%		%	
Current contraceptive use	49.1	54.2	48.1	52.0	48.2	54.9
Method mix, among users						
Male compliant	30.7	19.3	31.4	26.3	30.8	24.5
Female controlled	69.3	80.7	68.7	73.7	69.2	75.5
Covert contraceptive use	**9.5**	**35.0*****	9.4	15.5	10.9	13.0
**Kenya**						
	RC		Emotional IPV		Physical/ Sexual IPV	
	No RC (n=3135)	RC (n=251)	No IPV (n=2741)	IPV (n=646)	No IPV (n=2986)	IPV (n=401)
	%		%		%	
Current contraceptive use	77.9	74.2	77.2	79.2	77.8	76.3
Method mix, among users						
Male compliant	10.7	12.1	10.3	12.6	10.6	11.5
Female controlled	89.4	87.9	89.7	87.4	89.4	88.5
Covert contraceptive use	**5.6**	**30.8*****	**6.1**	**13.2*****	**6.1**	**17.5*****

p-value from bivariate logistic regression ±<0.1; *<0.05; **<0.01; ***<0.001; bolding represents significance p<0.05

For peer review only - http://bmjopen.bmj.com/site/about/guidelines.xhtml

Male compliant: Male sterilization, male condom, female condom, diaphragm, foam/jelly, standard days/cycle beads, rhythm, withdrawal, other traditional; Female-controlled: Female sterilization, implant, IUD, injectables, pills, EC, LAM

For peer review only

In multivariable models, RC remained consistently associated with increased covert use of contraception across sites (Burkina Faso: aOR=2.84; 95% CI=1.21-6.64; Côte d'Ivoire: aOR=4.45; 95% CI=1.76-11.25; Kenya: aOR=5.77; 95% CI=3.51-9.46; Table 4). Emotional IPV was associated further with covert contraceptive use in Burkina Faso (aOR=2.99; 95% CI=1.56-5.74) and physical/sexual IPV with covert contraceptive use in Kenya (aOR=2.35; 95% CI=1.33-4.17). In Kenya, emotional IPV was associated with increased odds of current contraceptive use (aOR=1.44; CI=1.04-2.01), and in Burkina Faso, emotional IPV was marginally associated with current contraceptive use below the significance threshold (aOR=1.36; 95% CI=0.97-1.92; p=0.08). Also, in Burkina Faso, RC experience was associated with non-significant decreases in current contraceptive use (aOR=0.66; 95% CI=0.43-1.01; p=0.06). None of the violence forms displayed significant associations with method type.

Table 4. Multivariable Logistic Regression between Past-Year RC and IPV and Contraceptive Use Outcomes Among Married/Partnered Women in Need of Contraception, per Country

		**Burkina Faso**	
	RC	Emotional IPV	Physical/ Sexual IPV
		aOR^{œ} (95% CI)	
Current contraceptive use	0.66 (0.43, 1.01)$^{\pm}$	1.36 (0.97, 1.92)$^{\pm}$	0.91 (0.54, 1.54)
Method mix, among users			
Male compliant	ref	ref	ref
Female controlled	0.81 (0.36, 1.86)	1.23 (0.72, 2.09)	1.14 (0.47, 2.73)
Covert contraceptive use	**2.84 (1.21, 6.64)***	**2.99 (1.56, 5.74)*****	0.83 (0.36, 1.91)
		**Cote d'Ivoire**	
	RC	Emotional IPV	Physical/ Sexual IPV
		aOR^{œ} (95% CI)	
Current contraceptive use	1.3 (0.72, 2.34)	1.15 (0.67, 1.97)	1.25 (0.61, 2.56)
Method mix, among users			
Male compliant	ref	ref	ref
Female controlled	--	1.2 (0.67, 2.14)	--
Covert contraceptive use	**4.45 (1.76, 11.25)****	1.88 (0.87, 4.05)	0.59 (0.19, 1.86)
		**Kenya**	
	RC	Emotional IPV	Physical/ Sexual IPV
		aOR^{œ} (95% CI)	
Current contraceptive use	0.84 (0.57, 1.23)	**1.44 (1.04, 2.01)***	0.82 (0.60, 1.13)
Method mix, among users			
Male compliant	ref	ref	ref
Female controlled	0.97 (0.55, 1.69)	**0.73 (0.50, 1.05)**$^{\pm}$	1.18 (0.67, 2.07)
Covert contraceptive use	**5.77 (3.51, 9.46)*****	0.8 (0.45, 1.42)	**2.35 (1.33, 4.17)****

$^{\pm}$<0.1; *<0.05; **<0.01; ***<0.001; bolding represents significance p<0.05
^{œ}aOR adjusted for other forms of violence, residence, education, parity, marital status, polygyny, employment
-- cells n<10 suppressed for regression
Male compliant: Male sterilization, male condom, female condom, diaphragm, foam/jelly, standard days/cycle beads, rhythm, withdrawal, other traditional; Female-controlled: Female sterilization, implant, IUD, injectables, pills, EC, LAM

Discussion

Leveraging large-scale, population-based samples, this analysis illuminates the prevalence of RC and overlap with physical/sexual and emotional IPV, and its impact on current and covert contraceptive use in Burkina Faso, Côte d'Ivoire, and Kenya. Data illustrate that for women experiencing RC who are in need of contraception, RC often occurred in isolation (31.7% in Burkina Faso to 45.8% in Côte d'Ivoire) or coupled with emotional IPV (16.7% in Côte d'Ivoire to 36.0% in Burkina Faso). Accordingly, only situating RC interventions within IPV programs and response services may overlook the needs of women who are experiencing this unique, yet detrimental, form of violence. Further, screening for physical or sexual IPV alone will fail to capture most RC experiences.

Notably, experience of past-year RC was consistently associated with increased covert use of contraception across sites (Burkina Faso: aOR=2.84; 95% CI=1.21-6.64; Côte d'Ivoire: aOR=4.45; 95% CI=1.76-11.25; Kenya: aOR=5.77; 95% CI=3.51-9.46), however, similar results were not observed for female-controlled method use. Covert use may prove a promising, yet risky safety strategy for women seeking to avert pregnancy and also experiencing RC.[12] Qualitative data with IPV and RC survivors in Nairobi indicated the cyclic covert use experiences of women—first electing to use contraception covertly given RC, then IPV or RC upon partner finding out about use, followed by a subsequent attempt to use a different contraceptive method covertly.[29] While providers may recommend female-controlled methods in light of partner disapproval, such as IUDs or implants, control of method use alone may not be enough to protect against unintended pregnancy when faced with RC. Instead, providers must be aware of RC and inquire about covert use preferences and potential RC experiences during contraceptive counseling to ensure method continuity and aversion of pregnancy without detriment to women's safety. Specifically, understanding previous experiences with side effects will be essential to ensure that women are able to use methods covertly. Further follow-up care will be necessary for women experiencing RC and covert use, as method switching may help minimize risk of partner suspicion.

Consistent with Demographic and Health Survey data, associations between physical/sexual IPV and contraceptive use were statistically insignificant,[17] however, emotional IPV was associated with significant increases in current contraceptive use in Kenya (aOR=1.44; 95% CI=1.04; p<0.05) and trend towards association in Burkina Faso (aOR=1.36; 95% CI=0.97-1.92; p=0.06). These results may signify important benchmarks on the abuse cycle and pathways to help-seeking—women wishing to avert pregnancy within violent relationships are acting on their reproductive preferences to utilize contraception. Further, they justify the inclusion of emotional abuse measures within large-scale surveys given its high prevalence and concurrence with RC.

Results must be considered in light of limitations. Foremost, findings are cross-sectional, limiting conclusions surrounding temporality of associations—longitudinal work is needed to disentangle whether women are using contraception because of experienced violence or whether the violence is incurred given contraceptive use. Further, while RC measures have

For peer review only - http://bmjopen.bmj.com/site/about/guidelines.xhtml

improved since this form of violence of first conceptualized, abortion coercion and forced contraceptive use are still not components of the RC scale, limiting our ability to capture a more inclusive range of RC experiences. Our RC measures also did not account for condom use manipulation and thus may undercount the true burden of RC in these contexts. Additionally, pregnant women were not included in the analytic sample, which could further underestimate RC prevalence as these women may be pregnant due to RC. However, including pregnant women in the sample would also lead to a systematic attenuation of the results between RC and contraceptive use given that these women are not in need of contraception. Women in dating partnerships were also excluded from the present analysis due to embedded survey skip logic, though evidence among urban adolescents and young women indicates high prevalence of RC;[30] further research and oversampling is needed to understand reproductive health needs for this high risk subpopulation. Social desirability biases and privacy concerns could further contribute to underreporting of experiences of violence, despite extensive training and privacy protocols aligned with best practices.[23,24]

Importantly, policymakers and family planning and violence service providers must recognize RC and its health impact to holistically support women's needs. Work to dismantle deep-rooted social norms that dictate spousal permission prior to contraceptive provision must be included in healthcare provider trainings on how to support women experiencing RC. Interventions such as Addressing Reproductive Coercion in Health Settings (ARCHES) in Kenya[31] and have proven useful in decreasing RC through training family planning service providers to recognize RC as a prevalent form of violence that impacts women's reproductive health[31]—scale-up of such programs may help prevent recurrent violence. Integration of violence and reproductive health prevention and response services can further guarantee that women's needs are met regardless of type of abuse incurred. Standardization of recommendations can further ensure that women are able to enact safety strategies to protect their reproductive health while concurrently mitigating the impact of abuse. Though many countries have made recent notable steps towards GBV prevention and response, national policies do not currently recognize RC. Policies must not only name RC as a detriment to women's health, but also include practical rights-based solutions that ensure women's privacy in contraceptive decision-making and freedom from partner interference, such as universal, affordable, and judgement-free provision of covert contraceptive methods and emergency contraception, to ultimately counteract its reproductive health impact.

For peer review only - http://bmjopen.bmj.com/site/about/guidelines.xhtml

Ethics approval statement

Institutional Review Board approval was obtained at JHSPH (00009677), Comité d'Ethique pour la recherche en santé and the Ministère de la Santé et Ministère de l'Enseignement Supérieur, de la Recherche Scientifique et de l'Innovation (ISSP/DA/GG/062/2021), Comité d'Ethique de la Recherche Institut Pasteur de la Côte d'Ivoire (150-21/MSHP/CNESVS-km), and Kenya Medical Research Institute (KEMRI) Ethics Review Committee (KNH-ERC/Mod&SAE/314).

Contributorship statement

SNW: study design, oversight, analysis, initial draft. HLT: analysis, graphics, initial draft. MT, GG, FB, YO, RM, RF, PG: study design, oversight of data collection, ethical adherence, interpretation, and editing. MRD: study design, oversight, initial draft. All authors participated in writing and approving the final manuscript.

Competing interests

None declared.

Funding

This work was supported, in whole, by the Bill & Melinda Gates Foundation [009639]. Under the grant conditions of the Foundation, a Creative Commons Attribution 4.0 Generic License has already been assigned to the Author Accepted Manuscript version that might arise from this submission.

Data availability statement

Data are available upon request from pmadata.org.

For peer review only

References

1. Sardinha L, Maheu-Giroux M, Stöckl H, Meyer SR, García-Moreno C. Global, regional, and national prevalence estimates of physical or sexual, or both, intimate partner violence against women in 2018. *Lancet*. 2022;399(10327):803-813. doi:10.1016/S0140-6736(21)02664-7
2. Stylianou AM. Economic abuse within intimate partner violence: A review of the literature. *Violence Vict*. 2018;33(1):3-22. doi:10.1891/0886-6708.VV-D-16-00112
3. Heise L, Pallitto C, García-Moreno C, Clark CJ. Measuring psychological abuse by intimate partners: Constructing a cross-cultural indicator for the Sustainable Development Goals. *SSM - Popul Heal*. 2019;9(September 2018):1-6. doi:10.1016/j.ssmph.2019.100377
4. Silverman JG, Raj A. Intimate Partner Violence and Reproductive Coercion : Global Barriers to Women's Reproductive Control. *Plos Med*. 2014;11(9):9-12. doi:10.1371/journal.pmed.1001723
5. Grace KT, Anderson JC. Reproductive Coercion. *Trauma, Violence, Abus*. Published online 2016:1-20. doi:10.1177/1524838016663935
6. Miller E, Decker MR, McCauley HL, et al. Pregnancy coercion, intimate partner violence and unintended pregnancy. *Contraception*. 2010;81(4):316-322. doi:10.1016/j.contraception.2009.12.004
7. Miller E, Decker MR, McCauley HL, et al. A family planning clinic partner violence intervention to reduce risk associated with reproductive coercion. *Contraception*. 2011;83(3):274-280. doi:10.1016/j.contraception.2010.07.013
8. Bergmann JN, Stockman JK. How does intimate partner violence affect condom and oral contraceptive use in the United States ? A systematic review of the literature. *Contraception*. 2015;91:438-455.
9. Tarzia L, Srinivasan S, Marino J, Hegarty K. Exploring the gray areas between "stealthing" and reproductive coercion and abuse. *Women Heal*. 2020;60(10):1174-1184. doi:10.1080/03630242.2020.1804517
10. Wood SN, Kennedy SR, Akumu I, et al. Reproductive Coercion among Intimate Partner Violence Survivors in Nairobi. *Stud Fam Plann*. 2020;51(4):343-360.
11. Wood SN, Dozier J, Karp C, et al. Pregnancy Coercion, Correlates, and Associated Modern Contraceptive Use Within a Nationally Representative Sample of Ethiopian Women. In: *Population Association of America Annual Meeting*. ; 2021.
12. Silverman JG, Challa S, Boyce SC, Averbach S, Raj A. Associations of reproductive coercion and intimate partner violence with overt and covert family planning use among married adolescent girls in Niger. *EClinicalMedicine*. 2020;22(100359).
13. Boyce SC, Uysal J, Delong SM, et al. Women's and girls' experiences of reproductive coercion and opportunities for intervention in family planning clinics in Nairobi, Kenya: A qualitative study. *Reprod Health*. 2020;17(1):1-12. doi:10.1186/s12978-020-00942-7
14. Darroch JE, Singh S. Trends in contraceptive need and use in developing countries in 2003, 2008, and 2012: An analysis of national surveys. *Lancet*. 2013;381(9879):1756-1762. doi:10.1016/S0140-6736(13)60597-8
15. Alkema L, Chou D, Hogan D, et al. Global, regional, and national levels and trends in maternal mortality between 1990 and 2015, with scenario-based projections to 2030: a systematic analysis by the UN Maternal Mortality Estimation Inter-Agency Group. *Lancet*. 2016;387(10017):462-474. doi:10.1016/S0140-6736(15)00838-7
16. Tsui AO, McDonald-Mosley R, Burke AE. Family planning and the burden of unintended

For peer review only

pregnancies. *Epidemiol Rev*. 2010;32(1):152-174. doi:10.1093/epirev/mxq012

17. Macquarrie KLD, Mallick L, Kishor S. *DHS Analytical Studies 57: Intimate Partner Violence and Interruption to Contraceptive Use*.; 2016.

18. Maxwell L, H B, Ndyanabo A, et al. The impact of intimate partner violence on women's contraceptive use: Evidence from the Rakai Community Cohort Study in Rakai, Uganda. *Soc Sci Med*. 2018;209(April):25-32. doi:10.1016/j.socscimed.2018.04.050 LK - http://vb3lk7eb4t.search.serialssolutions.com?sid=EMBASE&issn=18735347&id=doi:10.1016%2Fj.socscimed.2018.04.050&atitle=The+impact+of+intimate+partner+violence+on+women%27s+contraceptive+use%3A+Evidence+from+the+Rakai+Community+Cohort+Study+in+Rakai%2C+Uganda&stitle=Soc.+Sci.+Med.&title=Social+Science+and+Medicine&volume=209&issue=&spage=25&epage=32&aulast=Maxwell&aufirst=Lauren&auinit=L.&aufull=Maxwell+L.&coden=SSMDE&isbn=&pages=25-32&date=2018&auinit1=L&aui

19. United Nations Development Programme. *Human Development Report 2020: The Next Frontier Human Development and the Anthropocene: Burkina Faso*.; 2020.

20. United Nations Development Programme. *Human Development Report 2020: The Next Frontier Human Development and the Anthropocene: Cote d'Ivoire*.; 2020.

21. United Nations Development Programme. *Human Development Report 2020: The Next Frontier Human Development and the Anthropocene: Kenya*.; 2020.

22. United Nations. Ratification Status for CEDAW-Convention on the Elimination of All Forms of Discrimination against Women. UN Treaty Body Database. Published 2022. Accessed April 4, 2022. https://tbinternet.ohchr.org/_layouts/15/TreatyBodyExternal/Treaty.aspx?Treaty=CEDAW&Lang=en

23. WHO. *Putting Women First: Ethical and Safety Recommendations for Research on Domestic Violence Against Women*.; 1999.

24. World Health Organization (WHO). *Ethical and Safety Recommendations for Intervention Research on Violence against Women*.; 2016.

25. Straus M, Hamby S, Boney-McCoy S, Sugarman D. The Revised Conflict Tactics Scales (CTS2). *J Fam Issues*. 1996;17(3):283-316.

26. McCauley HL, Silverman JG, Jones KA, et al. Psychometric properties and refinement of the Reproductive Coercion Scale. *Contraception*. 2017;95(3):292-298. doi:10.1016/j.contraception.2016.09.010

27. DHS. Demographic and Health Surveys: Model Woman's Questionnaire. 2016;(1):1-75.

28. Choiriyyah I, Becker S. Measuring Women's Covert Use of Modern Contraception in Cross-Sectional Surveys. *Stud Fam Plann*. 2018;49(2):143-157. doi:10.1111/sifp.12053

29. Wood SN, Kennedy SR, Akumu I, et al. Understanding the link between reproductive coercion and covert use of contraception as a safety strategy for women experiencing violence in Nairobi's urban informal settlements. *Violence Against Women Accept Press*. Published online 2022.

30. Decker MR, Wood SN, Byrne ME, et al. Gendered power dynamics and threats to sexual and reproductive autonomy among adolescent girls and young adult women: A cross-sectional survey in three urban settings. *PLoS One*. 2021;16(11 November):1-16. doi:10.1371/journal.pone.0257009

31. Uysal J, Carter N, Johns N, et al. Protocol for a matched-pair cluster control trial of ARCHES (Addressing Reproductive Coercion in Health Settings) among women and girls

For peer review only

For peer review only - http://bmjopen.bmj.com/site/about/guidelines.xhtml

1 seeking contraceptive services from community-based clinics in Nairobi, Kenya. *Reprod Health*. 2020;17(1):1-14. doi:10.1186/s12978-020-00916-9

For peer review only

Figure 1. Venn Diagrams of IPV and Reproductive Coercion Experiences, Among Partnered Women with Need for Contraception, by Country

For peer review only

For peer review only - http://bmjopen.bmj.com/site/about/guidelines.xhtml

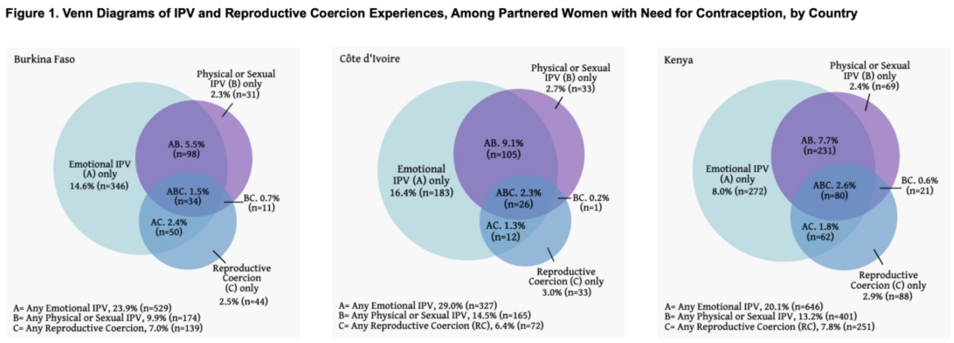

Venn Diagrams of IPV and Reproductive Coercion Experiences, Among Partnered Women with Need for Contraception, by Country

496x256mm (144 x 144 DPI)

STROBE Statement—Checklist of items that should be included in reports of ***cross-sectional studies***

	Item No	Recommendation	Page No
**Title and abstract**	1	(*a*) Indicate the study's design with a commonly used term in the title or the abstract	1
		(*b*) Provide in the abstract an informative and balanced summary of what was done and what was found	2
**Introduction**			
Background/rationale	2	Explain the scientific background and rationale for the investigation being reported	4
Objectives	3	State specific objectives, including any prespecified hypotheses	4
**Methods**			
Study design	4	Present key elements of study design early in the paper	
Setting	5	Describe the setting, locations, and relevant dates, including periods of recruitment, exposure, follow-up, and data collection	5
Participants	6	(*a*) Give the eligibility criteria, and the sources and methods of selection of participants	5
Variables	7	Clearly define all outcomes, exposures, predictors, potential confounders, and effect modifiers. Give diagnostic criteria, if applicable	6
Data sources/ measurement	8*	For each variable of interest, give sources of data and details of methods of assessment (measurement). Describe comparability of assessment methods if there is more than one group	*6*
Bias	9	Describe any efforts to address potential sources of bias	8
Study size	10	Explain how the study size was arrived at	5-6
Quantitative variables	11	Explain how quantitative variables were handled in the analyses. If applicable, describe which groupings were chosen and why	6
Statistical methods	12	(*a*) Describe all statistical methods, including those used to control for confounding	6
		(*b*) Describe any methods used to examine subgroups and interactions	NA
		(*c*) Explain how missing data were addressed	6
		(*d*) If applicable, describe analytical methods taking account of sampling strategy	NA
		(*e*) Describe any sensitivity analyses	NA
**Results**			
Participants	13*	(a) Report numbers of individuals at each stage of study—eg numbers potentially eligible, examined for eligibility, confirmed eligible, included in the study, completing follow-up, and analysed	5-6
		(b) Give reasons for non-participation at each stage	5-6
		(c) Consider use of a flow diagram	NA
Descriptive data	14*	(a) Give characteristics of study participants (eg demographic, clinical, social) and information on exposures and potential confounders	7
		(b) Indicate number of participants with missing data for each variable of interest	6
Outcome data	15*	Report numbers of outcome events or summary measures	7
Main results	16	(*a*) Give unadjusted estimates and, if applicable, confounder-adjusted estimates and their precision (eg, 95% confidence interval). Make clear which confounders were adjusted for and why they were included	7

For peer review only - http://bmjopen.bmj.com/site/about/guidelines.xhtml

		(*b*) Report category boundaries when continuous variables were categorized	NA
		(*c*) If relevant, consider translating estimates of relative risk into absolute risk for a meaningful time period	NA
Other analyses	17	Report other analyses done—eg analyses of subgroups and interactions, and sensitivity analyses	7
**Discussion**			
Key results	18	Summarise key results with reference to study objectives	7-8
Limitations	19	Discuss limitations of the study, taking into account sources of potential bias or imprecision. Discuss both direction and magnitude of any potential bias	8
Interpretation	20	Give a cautious overall interpretation of results considering objectives, limitations, multiplicity of analyses, results from similar studies, and other relevant evidence	8
Generalisability	21	Discuss the generalisability (external validity) of the study results	8
**Other information**			
Funding	22	Give the source of funding and the role of the funders for the present study and, if applicable, for the original study on which the present article is based	In system

*Give information separately for exposed and unexposed groups.

Note: An Explanation and Elaboration article discusses each checklist item and gives methodological background and published examples of transparent reporting. The STROBE checklist is best used in conjunction with this article (freely available on the Web sites of PLoS Medicine at http://www.plosmedicine.org/, Annals of Internal Medicine at http://www.annals.org/, and Epidemiology at http://www.epidem.com/). Information on the STROBE Initiative is available at www.strobe-statement.org.

For peer review only
